# Genome-wide identification, characterization, and expression analyses of the solute carrier 2 (*SLC2*) gene family in fall armyworm (*Spodoptera frugiperda*)

**DOI:** 10.1186/s12864-025-12336-9

**Published:** 2026-01-23

**Authors:** Hala M. Zoghly, Ahmed M. K. Ghallab, Mayada M. Emam, Mayar A. Abdelbaky, Mohamed A. Rashed, Walaa El-Sayed, Eman M. Abdelmaksoud

**Affiliations:** 1 Faculty of Agriculture Department of Genetics, Ain Shams University, Hadayek Shoubra, P.O. Box 68, Cairo, 11241 Egypt; 2 Faculty of Agriculture Biotechnology Program, Ain Shams University, Hadayek Shoubra, P.O. Box 68, Cairo, 11241 Egypt; 3 Faculty of Agriculture Department of Plant Protection, Ain Shams University, Hadayek Shubra, P.O. Box 68, Cairo, Egypt

**Keywords:** *Spodoptera frugiperda*, Major facilitator superfamily (MFS), Solute carrier 2 (SLC2), Genome-wide identification, Trehalose transporters (TRETs), Gene expression analysis, Emamectin benzoate (EMB), Spinosad (SPD), Acetone (Ac)

## Abstract

The solute carrier 2 (*SLC2*) gene family, responsible for hexose and polyol transport, has not been fully characterized in *Spodoptera frugiperda*, a globally invasive pest. Among this family, the trehalose transporter (*TRET*) genes, the largest and most functionally important subfamily, contribute to energy homeostasis and stress adaptation in insects. Despite their importance, *TRET* genes’ role in insect responses to bioinsecticide exposure remains largely unknown. Therefore, this study aimed to identify and characterize *SLC2* genes, from which *TRET* genes were validated under bioinsecticidal stress in *S. frugiperda.* A genome-wide identification of the *SLC2* gene family in *S. frugiperda* was performed using bioinformatics approaches. Third-instar larvae were treated under controlled temperature and light conditions with the technical grade of Emamectin benzoate (EMB) and Spinosad (SPD), along with their solvent acetone (Ac) and negative control (NC). In parallel, field bioassays were conducted using the commercial formulations of both insecticides on second- and fourth-instar larvae. Gene expression levels of selected *TRET* genes were evaluated using qRT-PCR. A total of 86 putative SfruSLC2 members were identified and analyzed for their structural, functional, and physicochemical properties, confirming their stability as membrane-associated transporters. Laboratory treatments induced dose-dependent toxic symptoms, whereas field applications resulted in 100% mortality within 24 h. qRT-PCR results revealed significant upregulation of *TRET* genes, with EMB eliciting the highest expression, followed by SPD and Ac. EMB and Ac showed non-linear responses, while SPD induced a more linear pattern. This study provides the first genome-wide characterization of the *SLC2* gene family in *S. frugiperda* and highlights *TRET* genes as key mediators of energy regulation under bioinsecticide and Ac stress. These findings contribute novel insights into the molecular response mechanisms of *S. frugiperda* and may support future strategies for sustainable pest control and resistance management.

**Supplementary Information:**

The online version contains supplementary material available at 10.1186/s12864-025-12336-9.

## Introduction

The solute carrier (SLC) superfamily is one of the largest and most conserved transporter families across living organisms, indicating its importance for survival and reproduction [1, 2]. It encodes membrane proteins that mediate the transport of sugars, amino acids, nucleotides, ions, and drugs across cell membranes [3]. According to the Human Gene Nomenclature Committee (HGNC), SLCs are classified into 66 groups within three main Pfam clans: the Major Facilitator Superfamily (MFS), Acid-Polyamine-Organic Cation (APC), and Cation Proton Antiporter (CPA)/Anion Transporter (AT) superfamilies [2, 3]. Among them, MFS proteins such as SLC2 (facilitated hexose and polyol transporters), SLC17 (organic anion transporters), SLC22 (organic cation, zwitterions/cation, and organic anion transporters), and other SLCs function as facilitators, symporters, or antiporters, transporting a wide range of substrates essential for cellular homeostasis and metabolism [3–6].

The *SLC2* gene family encodes transporter proteins that facilitate hexose and polyol movement across membranes [3, 7]. In insects, it is divided into two major subfamilies: facilitated trehalose transporters (TRETs) and facilitated glucose transporters (GLUTs). These transporters, particularly in lepidopterans, mediate the transmembrane diffusion of sugars such as trehalose, glucose, and fructose [2, 7, 8]. In humans, SLC2 members (GLUTs) comprise 14 proteins classified into three groups (Classes I–III) based on sequence similarity, responsible for transporting glucose, galactose, fructose, and myoinositol [9, 10].

TRETs act as passive transporters essential for energy homeostasis by transferring trehalose from the fat body to the hemolymph and other energy-demanding tissues [11, 12]. The facilitated trehalose transporter (*Tret1*) gene is mainly expressed in the fat body, while the facilitated trehalose transporter-like (*Tret1-like or Tret2*) predominates in the Malpighian tubules, facilitating reciprocal trehalose transport [13, 14]. TRETs are highly diversified in polyphagous insects, likely to enhance their adaptability [12]. Although TRETs differ from GLUTs in substrate affinity and kinetics [11], they share structural similarity with mammalian class III GLUTs, especially GLUT6 and GLUT8 [7].

Trehalose, a glucose alternative, plays a crucial role in insect growth, development, molting, metamorphosis, and stress tolerance; hence, it is often called insect blood sugar [12, 15, 16]. Studies on *TRET* genes have been conducted in various insect species, including *Helicoverpa armigera*, *Bombyx mori*, *Nilaparvata lugens*, *Apis mellifera*, and *Drosophila melanogaster* [12]. These investigations revealed that insect species with broader host ranges and stronger adaptive capacities typically harbor multiple *TRET* variants, which contribute to the regulation of trehalose homeostasis under diverse stress conditions. In contrast, research on *TRET* genes in *S. frugiperda* remains scarce, being mainly restricted to the inhibition of trehalose synthesis and catabolism using RNA interference and trehalose inhibitors as potential management strategies [16, 17].

*S. frugiperda* (Fall armyworm) is a highly invasive lepidopteran pest that attacks over 350 plant species, including approximately 80 economically important crops [18, 19]. Originating from tropical and subtropical regions of America, *S. frugiperda* has spread worldwide through its exceptional migratory capacity [16]. The species was first detected in Egypt in May 2019, infesting maize fields in Kom Ombo, Aswan Governorate [20]. The larval stage is the most destructive, feeding on both vegetative and reproductive plant tissues, particularly those of the Poaceae family, and can cause yield losses of up to 80% under severe infestations [21–23]. Its devastating impact is further reinforced by a high reproductive potential (1,500–2,000 eggs per female) and resistance to nearly 40 insecticides [24–26].

Given the substantial damage and considerable economic impact caused by *S. frugiperda*, a wide range of chemical and biological control strategies have been employed. The extensive reliance on synthetic insecticides has led to serious challenges, including environmental contamination and the rapid development of resistance, limiting their long-term effectiveness. Therefore, developing green and sustainable pest management strategies is now essential [27]. Biopesticides are increasingly preferred for controlling this highly polyphagous pest because they are environmentally friendly, persistent, effective against multiple developmental stages, and pose minimal risks to non-target organisms, including human and beneficial insects [18, 28]. Notable examples include EMB and SPD [29, 30].

EMB (4″-epi-methylamino-4″-deoxyavermectin B1) is a semi-synthetic derivative of abamectin B1 with potent activity against a wide range of lepidopteran pests. Beyond its lethal effects, sublethal concentrations can alter insect development, behavior, fertility, and reproduction [30, 31]. Its mode of action involves increasing chloride ion influx by activating γ-aminobutyric acid (GABA) receptors and glutamate-gated chloride channels (GluCls), leading to neural dysfunction and death [31]. Additionally, EMB has been reported to induce apoptosis and DNA damage in *S. frugiperda* [31].

SPD, another bioinsecticide, is a natural mixture of two macrocyclic lactones, spinosyn A and spinosyn D, produced by the actinomycete *Saccharopolyspora spinosa* through fermentation [32]. It acts mainly on nicotinic acetylcholine receptors (nAChRs) and secondarily on GABA-gated chloride channels, causing neuronal overstimulation, muscle spasms, paralysis, and eventual death [32–35]. Sublethal exposure can disrupt larval hormonal balance, delay development, reduce body weight, deform pupae, and decrease adult emergence [29]. Despite its potency, SPD exhibits low toxicity to mammals and minimal effects on non-target insects [32, 35].

Therefore, this study aimed to identify, characterize, and functionally analyze the *SLC2* gene family in *S. frugiperda*, one of the most destructive insect pests worldwide, and to validate the expression of *TRET* genes under exposure to different concentrations of EMB and SPD bioinsecticides, as well as Ac as their solvent control. These genes were selected to test the hypothesis that *SLC2* subfamily genes, particularly *TRETs*, may respond to bioinsecticide exposure, indicating potential alterations in the insect’s energy regulation. This could provide new insights into *S. frugiperda* responses beyond the well-known neurotoxic effects of such compounds.

## Results

### Identification of *S. frugiperda *MFS (SfruMFS) members

The HMMER search resulted in 446 members. After subsequent filtering using InterPro and CD-Hit searches, 267 putative SfruMFS proteins were retained. This curated set of sequences was locally aligned with the *Drosophila melanogaster* MFS protein sequences set using BLASTp, and the results confirmed the similarity between the two sets with E-value < 1e − 5 and sequence identity > 40% (data not shown).

### *S. frugiperda* SLC2 (SfruSLC2) phylogeny and characterization

**Fig. 1:**
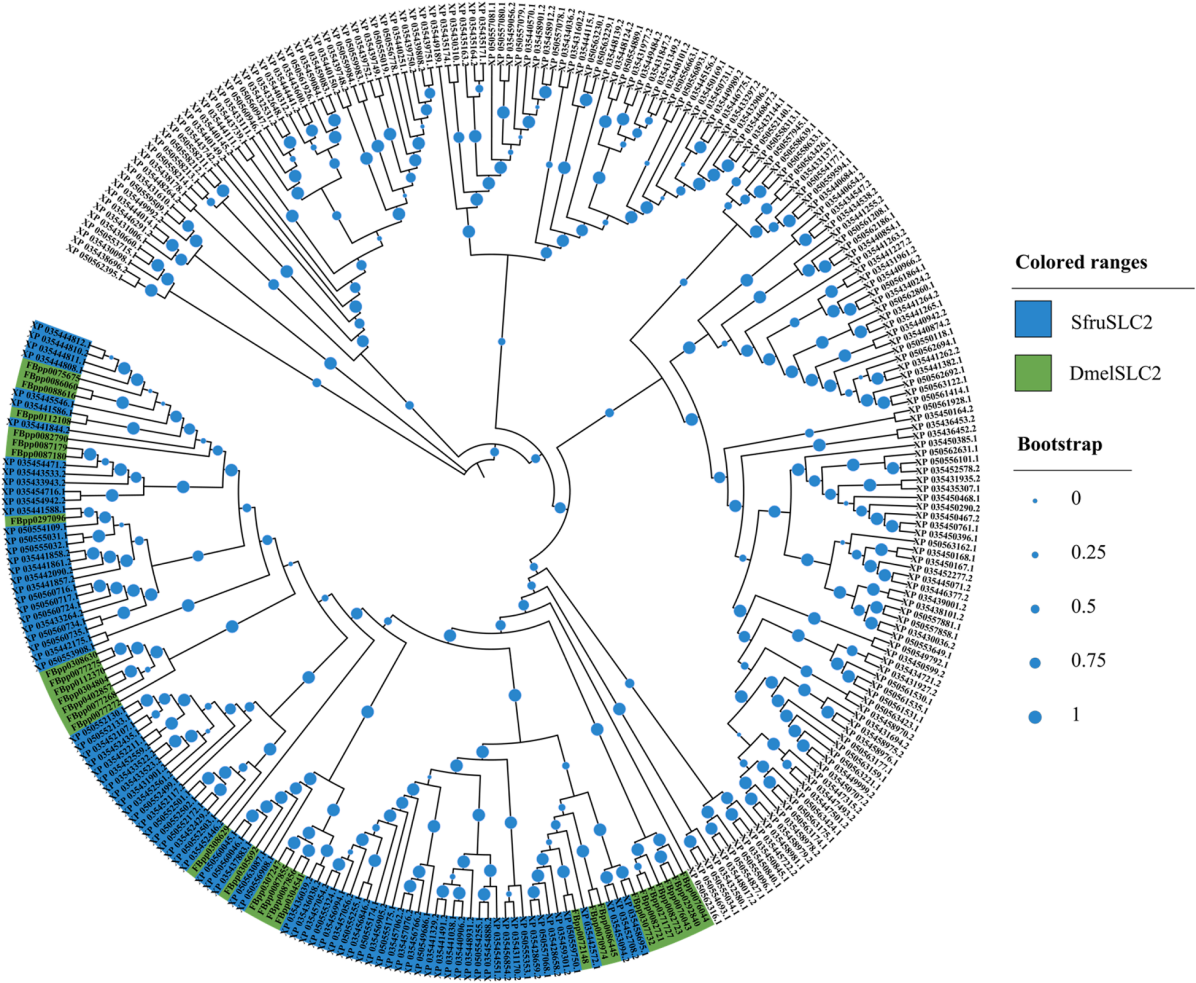
FastTree NJ phylogenetic tree of 267 putative *S. frugiperda* MFS members aligned with *D. melanogaster* SLC2 members. *D. melanogaster* SLC2 (DmelSLC2) members were colored green, while *S. frugiperda* MFS members, included in the same subclade of *D. melanogaster* SLC2, were colored blue and renamed as *S. frugiperda* SLC2 (SfruSLC2)

**Fig. 2:**
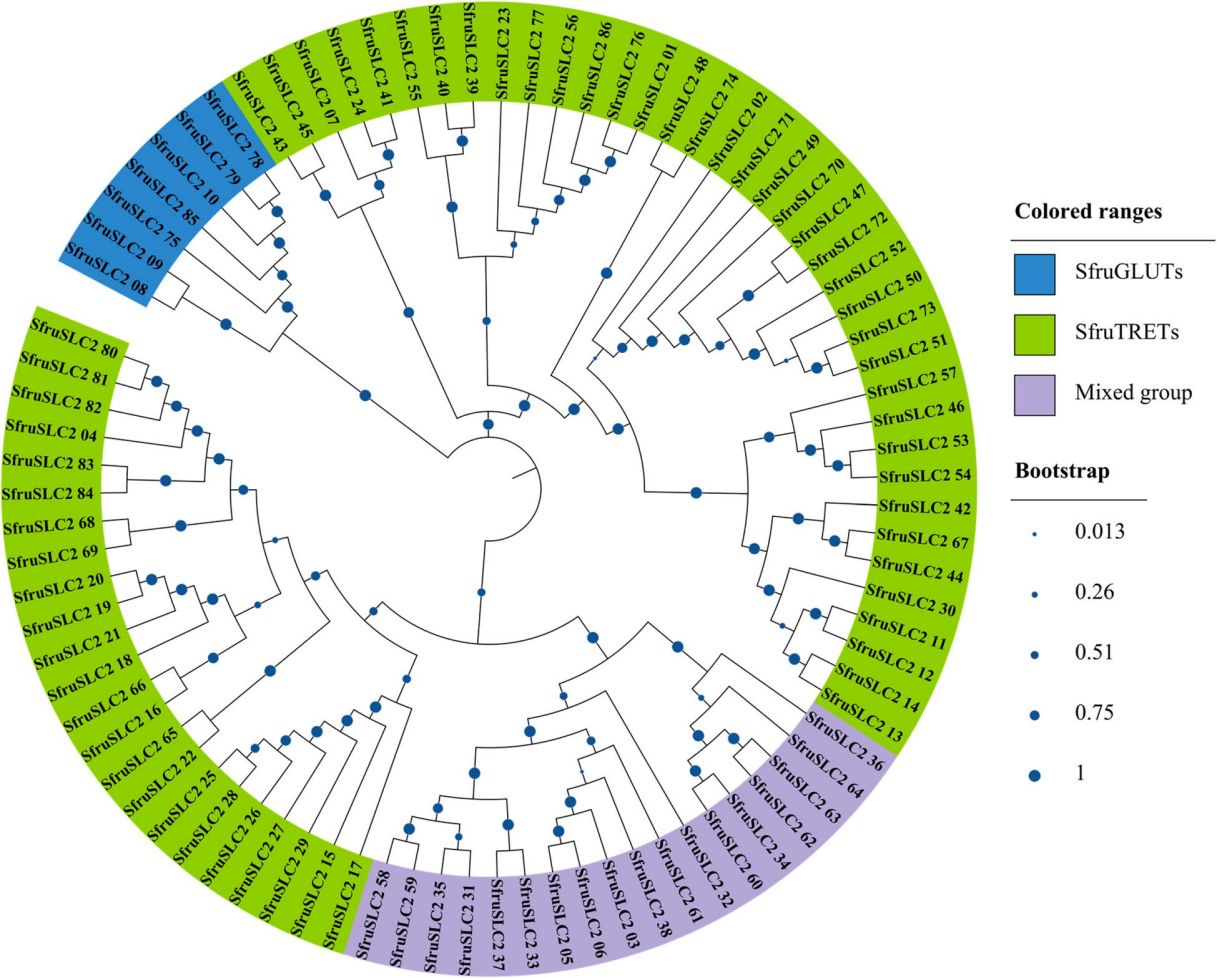
NJ phylogenetic tree of identified *S. frugiperda* SLC2 members, which were classified into three clads. Blue colored sequences represented the *S. frugiperda* GLUTs (SfruGLUTs), green colored members represented SfruTRETs, and purple-colored members represented mixed sequences whose majority were potential TRETs

**Fig. 3:**
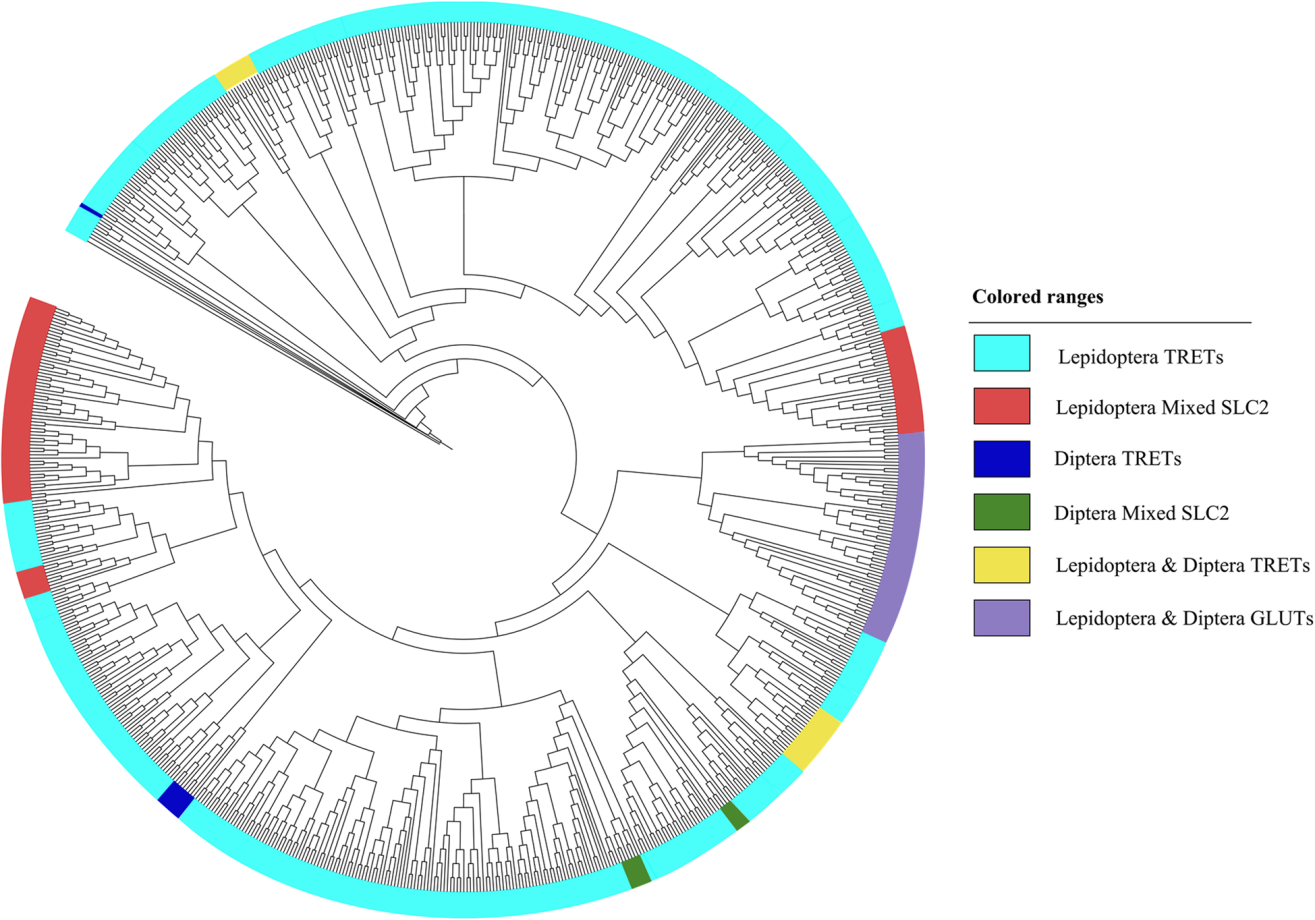
Maximum likelihood phylogenetic tree of orthologues SLC2 members in lepidopteran and non-lepidopteran insects. The light aqua-colored members are the largest Lepidopteran TRETs group (592 members). The blue-colored members are Dipteran TRETs (9 members). The yellow-colored group includes 24 Lepidopteran and 5 Dipteran TRETs. The purple group comprised 56 Lepidopteran and 5 Dipteran GLUTs. The green group included 10 dipteran mixed members containing TRETs and GLUTs. The red-colored group encompasses 98 Lepidopteran mixed members

A total of 267 putative SfruMFS members were aligned with 31 *Drosophila melanogaster* SLC2 (DmelSLC2) proteins for phylogenetic analysis (Fig. 1; Supplementary Table S1). The resulting tree identified 86 proteins (colored in blue) clustering with DmelSLC2, which were designated as verified SfruSLC2. These were grouped into three main clades (Fig. 2): seven GLUTs, 38 TRETs, and 41 mixed members (23 TRETs and 18 undefined but TRET-like according to their bootstrap high value and domain search). Cross-species comparison (Fig. 3) further confirmed the diversification of SfruSLC2 into similar clades across insects. The largest cluster was Lepidopteran TRETs (592 members), followed by smaller groups of Lepidopteran mixed (98), Lepidopteran and Dipteran GLUTs (61), Lepidopteran and Dipteran TRETs (29), and Dipteran TRETs (9), indicating evolutionary expansion and diversification of the SLC2 family in Lepidoptera.

Genomic and physicochemical characteristics of the identified SfruSLC2 proteins are summarized in Supplementary Tables S2 and S3. Protein lengths ranged from 174 amino acids to 782 amino acids, with an average of 502 amino acids, while their molecular weight (MW) ranged from ~ 18.9 to ~ 87.8 kDa with a mean of ~ 55.3 kDa. Theoretical isoelectric points (pI) varied between 5.04 and 9.66 (average 8.4), with most proteins (86%) exhibiting basic pI values (> 7). The Grand Average of Hydropathy (GRAVY) values were positive for 97.7% of proteins (average 0.502), indicating predominant hydrophobicity. Aliphatic index (AI) values were high (85.08–125.83; mean 109), suggesting good thermostability. Instability index (II) values ranged from 24.33 to 52.81 (mean 38.56), with 50 proteins classified as potentially stable (II < 40).

### Domain architecture and conserved motifs of SfruSLC2 proteins

**Fig. 4:**
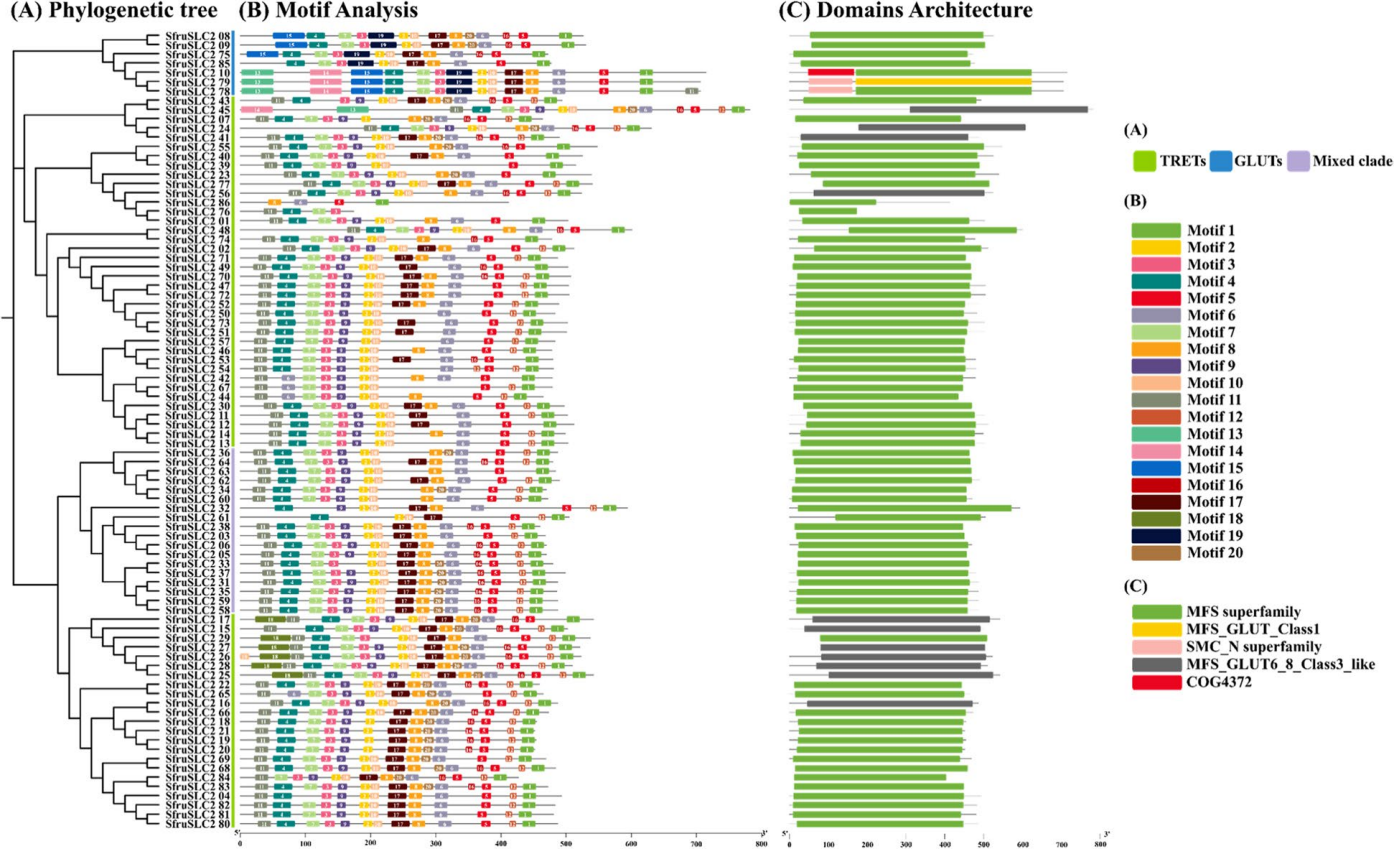
Neighbor-joining phylogenetic tree of SfruSLC2 members **A**, Graphical illustration of 20 conserved motifs distributed across SfruSLC2s proteins **B** and Domain architecture detected for SfruSLC2 **C**

The domain architectures of SfruSLC2 proteins are shown in Fig. 4C. The NCBI Conserved Domain Database (CDD) search identified three MFS domains: the major MFS superfamily (cl28910), MFS_GLUT_GLUT6_8_Class3_like (cd17358), and MFS_GLUT_Class1 (cd17431). The MFS superfamily domain was the most prevalent, present in 74 proteins, whereas the other two appeared in 11 and one protein, respectively. Additionally, two non-MFS domains were detected in SfruSLC2_10, SfruSLC2_78, and SfruSLC2_79.

**Fig. 5:**
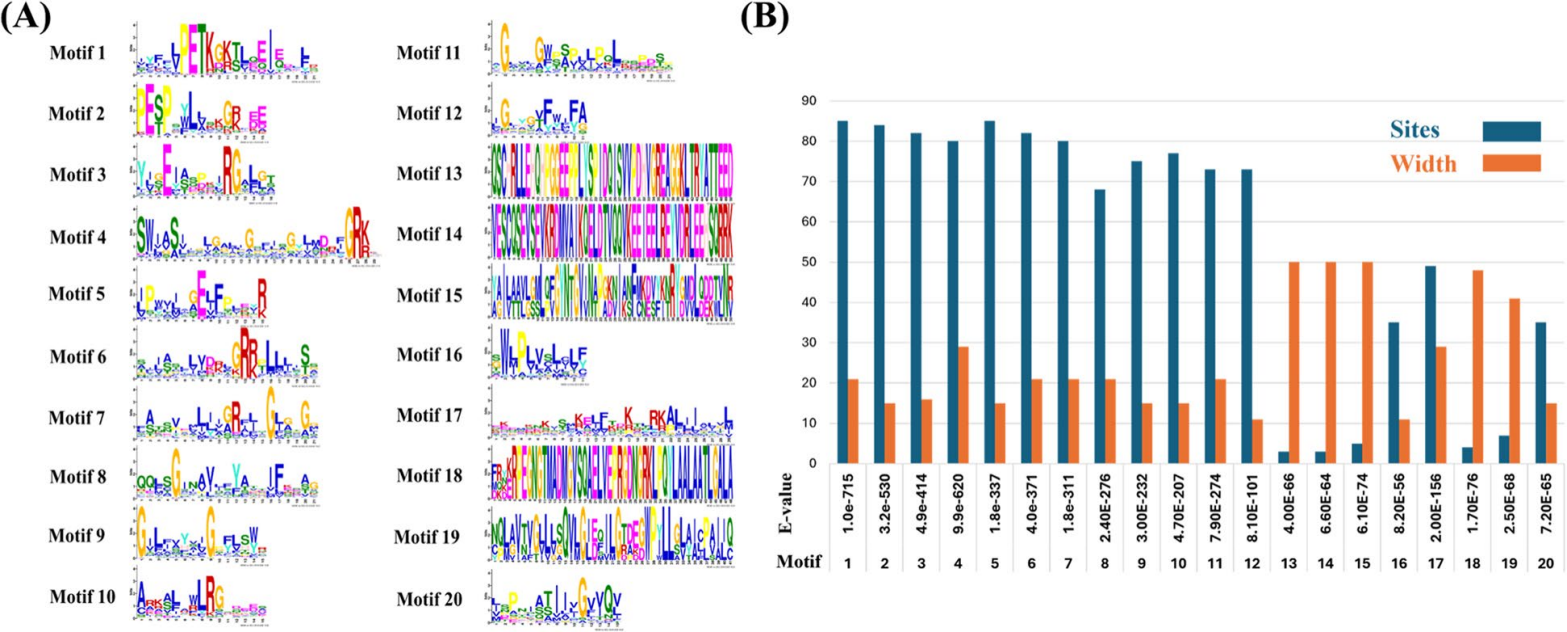
Sequence logos of the 20 conserved motifs identified in SfruSLC2 proteins (**A**), and graphical chart showing motif E-values, widths, and distribution sites (**B**)

The motifs distribution across the putative SfruSLC2 proteins is shown in Fig. 4B, with motif numbers and sequence logos presented in Fig. 5A, and their e-values, widths, and positions detailed in Fig. 5B. Motifs varied in length from 11 amino acids (motifs 12 and 16) to 50 amino acids (motifs 13–15), with an average width of ~ 26 aa. Motifs 1 and 5 were the most conserved, present in all sequences except SfruSLC2_76, where motif 1 occurred last. In contrast, motifs 13 and 14 were rare, found only in SfruSLC2_10, SfruSLC2_45, SfruSLC2_78, and SfruSLC2_79. SfruSLC2_76 and SfruSLC2_86 had the fewest motifs, containing only four each.

The SfruSLC2 sequences were arranged according to their phylogenetic tree (Fig. 4A). SfruSLC2 members were classified into three groups: GLUTs (blue), TRETs (green), and mixed members (purple). The GLUT group shared a highly conserved motif pattern (motifs 15, 4, 7, 3, 19, 2, 10, 17, 8, 20, 6, 16, 5, 1), with minor variations in SfruSLC2_75 and SfruSLC2_85, which lost some motifs. SfruSLC2_10, SfruSLC2_79, and SfruSLC2_78 showed strong similarity, but gained additional motifs (13, 14, and 11).

The TRETs formed two clades with a typical motif pattern (11, 4, 7, 3, 9, 2, 10, 17, 8, 20, 6, 16, 5, 12, 1), though motif 20 was frequently lost, while motifs 11, 5, and 1 were lost once. SfruSLC2_76 and SfruSLC2_86 contained only four motifs, correlating with shorter domain lengths. Several TRETs exhibited motif duplications or replacements, such as motif 6 replacing motif 4 in SfruSLC2_42, SfruSLC2_67, and SfruSLC2_44. SfruSLC2_48 exhibited a shift of motif location toward the C-terminal region, resulting in a corresponding shift in domain location. The second TRET clade uniquely included motif 18 in six members (SfruSLC2_17, SfruSLC2_29, SfruSLC2_27, SfruSLC2_26, SfruSLC2_28, SfruSLC2_25) and additional motif 10 duplication in SfruSLC2_26. SfruSLC2_65 had a duplication of motif 6 replacing motif 4, while SfruSLC2_84 experienced a clear deletion, resulting in the loss of motif 4.

The purple mixed group, included in the second TRET clade, showed similar motifs and domain structures to TRETs, supporting their classification within the same subfamily. Notably, SfruSLC2_32 and SfruSLC2_61 exhibited significant motif loss and shifts.

### SfruSLC2 subcellular localization, transmembrane, and signal peptide

**Fig. 6:**
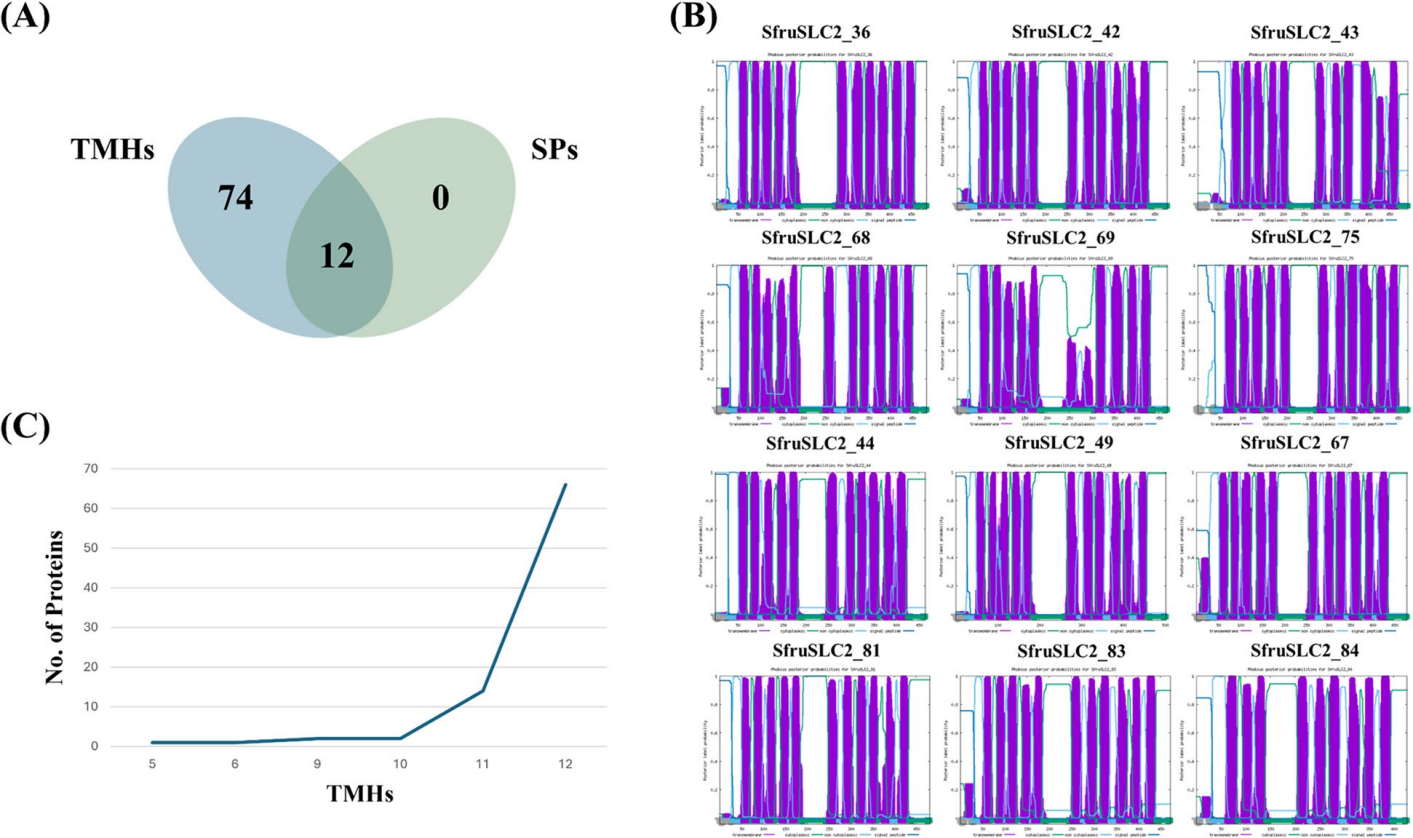
Venn diagram of SfruSLC2 signal peptides (SPs) and transmembrane helices (TMHs) domains (**A**), The 12 SfruSLC2 proteins that possessed N-terminal SPs (**B**), Histogram of the SfruSLC2 TMHs numbers (**C**)

The predicted subcellular localization of the 86 SfruSLC2 proteins was mainly at the plasma membrane, followed by the endoplasmic reticulum (Supplementary Figure S1). Twenty-five proteins were exclusively membrane-bound, while others showed multiple localizations. SfruSLC2_23 was the most widely distributed across five compartments, including the plasma membrane, mitochondria, cytoplasm, peroxisomes, and nucleus. Three members (SfruSLC2_29, 37, 51) were mitochondrial, three (SfruSLC2_19, 35, 71) were associated with lysosomes, and ten were extracellular (with SfruSLC2_48 being the most abundant). Prediction of transmembrane helices (TMHs) and signal peptides (SPs) in SfruSLC2 proteins revealed that 85 members contained TMHs, while only 12 possessed N-terminal SPs (SfruSLC2_36, 42, 43, 44, 49, 67, 68, 69, 75, 81, 83, and 84) (Fig. 6A–B). SP cleavage sites began at amino acid 1 (a. a. 1), extending to positions ranging from a.a. 22 in SfruSLC2_36 to a.a. 63 in SfruSLC2_43.

The number of TMHs in SfruSLC2 proteins ranged from 5 to 12, forming six groups (Fig. 6C). SfruSLC2_76 contained the fewest TMHs (5), followed by SfruSLC2_86 (6), SfruSLC2_69 and SfruSLC2_84 (9), and SfruSLC2_37 and SfruSLC2_50 (10). Fourteen proteins possessed 11 TMHs, while the remaining 66 members had 12 TMHs, the most common structure among the family.

### SfruSLC2 secondary and tertiary structure

**Fig.7:**
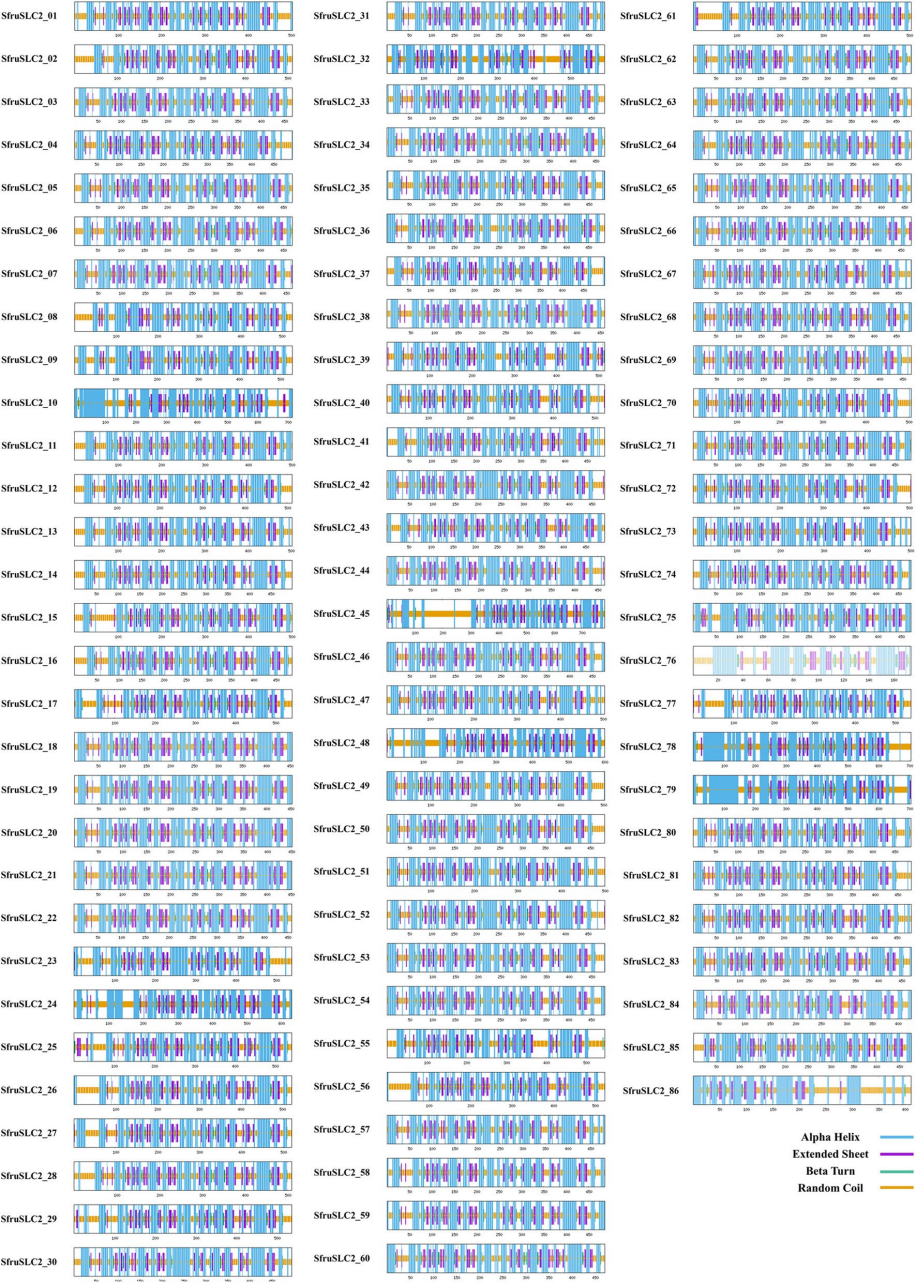
Secondary structure of SfruSL2 proteins

Secondary structure analysis (Supplementary Table S4; Fig. 7) revealed four components: α-helix, extended strand, β-turn, and random coil. The α-helix was dominant, averaging 48.74% and ranging from 37.72% (SfruSLC2_45) to 57.22% (SfruSLC2_79). The remaining elements followed the order: random coil (30.64%) > extended strand (15.64%) > β-turn (4.99%). SfruSLC2_45 exhibited the highest random coil content (46.93%), while SfruSLC2_20 had the lowest (25.44%). SfruSLC2_10 showed the smallest β-turn proportion (2.66%). Based on α-helix percentages, SfruSLC2 members were grouped into 14 similarity clusters.

Tertiary structure analysis of SfruSLC2 proteins (Supplementary Figure S2) revealed that α-helices (spiral regions) were the predominant structural component, consistent with their transmembrane nature. The QMEANBrane Z-scores of the generated models ranged from −5.608 to −0.546, with an average of −1.994 (Supplementary Table S5), indicating acceptable overall model quality for membrane proteins, confirming that the predicted conformations are consistent with typical structural features of membrane proteins. All SfruSLC2 members contained transmembrane regions except SfruSLC2_45. The hydrophobicity-based color scheme showed predominantly red helices, indicating that these proteins are largely hydrophobic. Overall, the tertiary structure results confirmed the secondary structure and TMH predictions.

### Short conserved motif (SCM) of SfruSLC2

**Fig.8:**
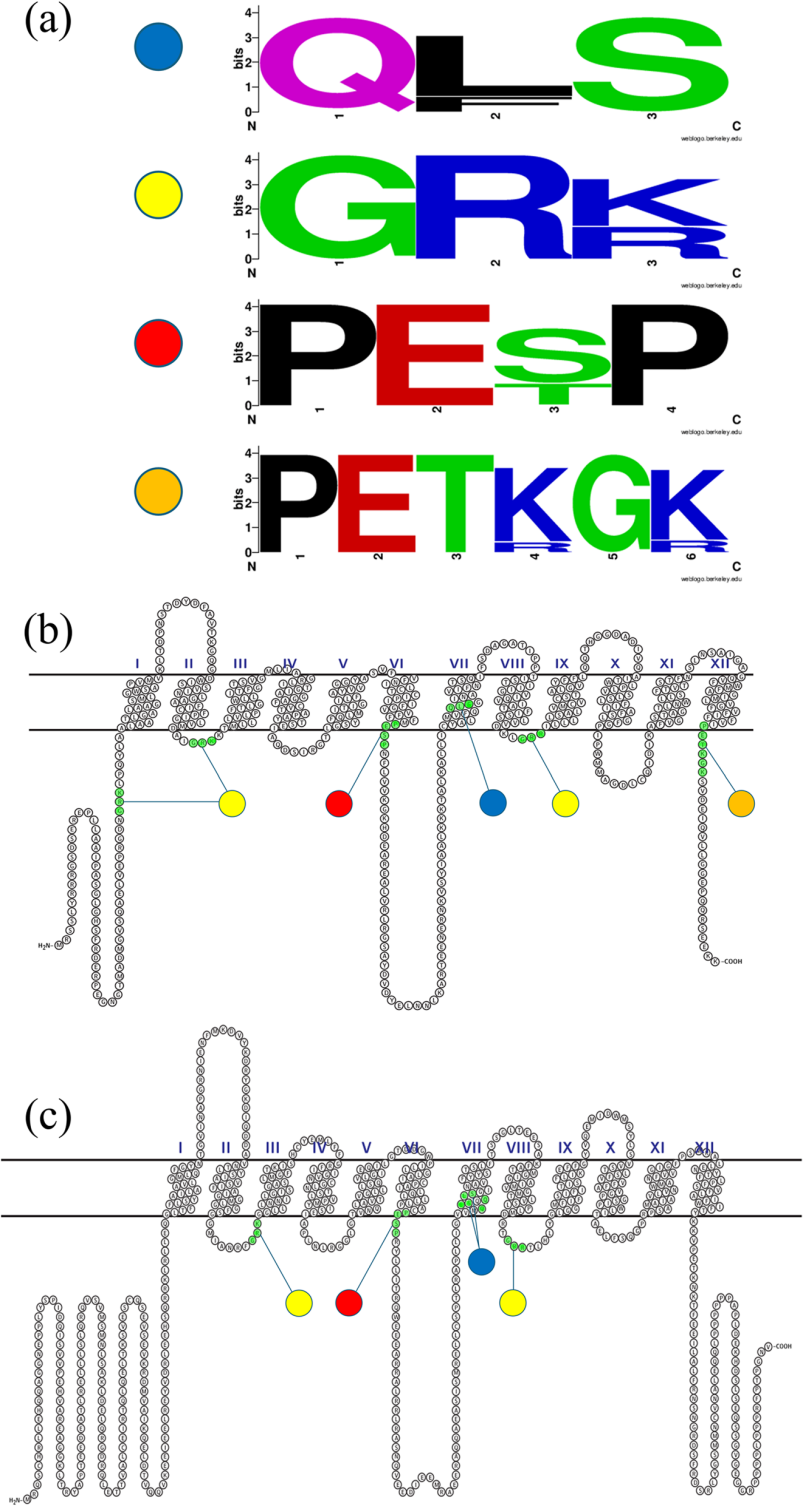
SfruSLC2 function–related conserved motifs analysis; logos of QL/FS, PES/TP, GRR/K, PET, and PETK/RGK/R motifs **a**, TRETs representative protein, SfruSLC2_07 **b**, and GLUTs representative protein, SfruSLC2_78 **c**

SCM analysis of SfruSLC2 proteins (Supplementary Table S6; Fig. 8a–c) revealed several characteristic motifs. The QL/FS motif appeared 29 times in 22 members, mainly in transmembrane helix 7, except in SfruSLC2_46, SfruSLC2_71, and SfruSLC2_76. GLUT-type proteins contained only the QLS motif, while TRETs possessed QL/FS motifs. The GRR/K motif was most abundant, detected 150 times across almost all members (except SfruSLC2_49 and SfruSLC2_61), mainly within loops 2 and 8; GRR occurred in 48 proteins and GRK in 62 TRETs, with 26 members harboring both. The PES/TP motif appeared in 71 proteins, represented as PESP in 51 members and PETP in 20 TRETs. Additionally, a single PETK/RGK/R motif was found at the C-terminus of 40 TRETs.

### Gene ontology and protein–protein interaction of SfruSLC2

**Fig.9:**
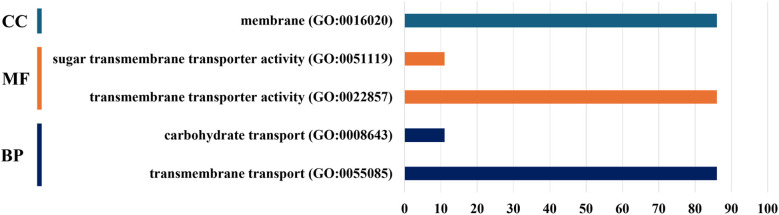
Histogram of the Gene Ontology (GO) prediction for SfruSLC2, showing the cellular component (CC), molecular function (MF), and biological process (BP)

Gene Ontology (GO) analysis of SfruSLC2 proteins (Fig. 9) revealed uniform functional annotations across all members. For biological process (BP), all proteins were associated with transmembrane transport (GO:0055085), while 11 members additionally participated in carbohydrate transport (GO:0008643). The molecular function (MF) was transmembrane transporter activity (GO:0022857) for all, with sugar transmembrane transporter activity (GO:0051119) restricted to the same 11 proteins. All SfruSLC2s were localized to the membrane (GO:0016020) as their cellular component (CC).

**Fig. 10:**
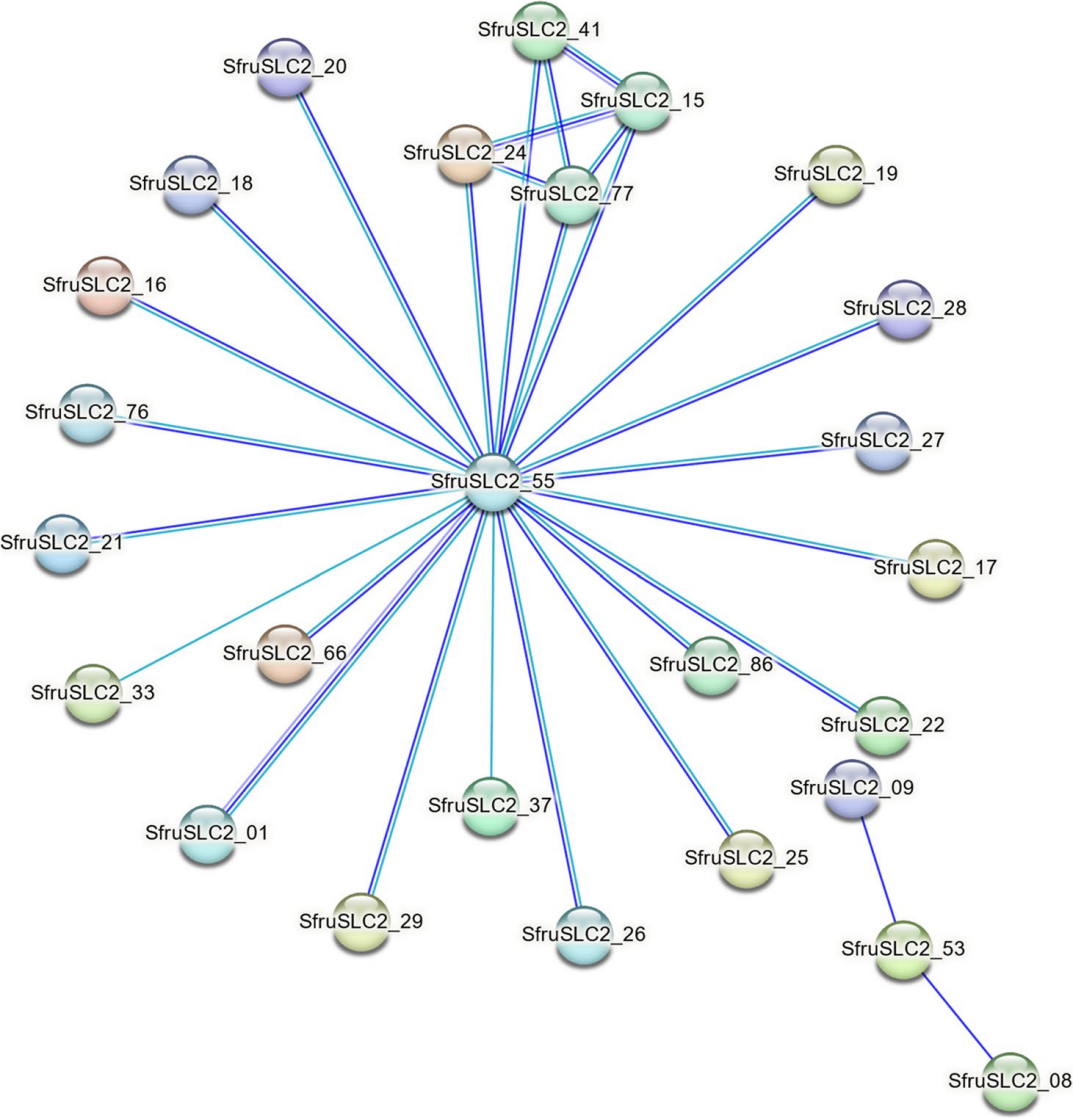
Protein–Protein interaction network of SLC2 proteins in *S. frugiperda*

The protein–protein interaction (PPI) network (Fig. 10) included 26 nodes and 29 edges, illustrating the interconnections among SfruSLC2 proteins. Blue nodes represented proteins with an identity > 96%, while red nodes had < 96%. SfruSLC2_55 was identified as the central hub, interacting with all proteins except SfruSLC2_08, SfruSLC2_09, and SfruSLC2_53, which showed only limited connections. A smaller internal cluster was observed among SfruSLC2_15, SfruSLC2_24, SfruSLC2_41, and SfruSLC2_77, while the remaining proteins exhibited single interactions with the core SfruSLC2_55.

### Chromosomal localization and gene structure of *SfruSLC2* genes

**Fig. 11:**
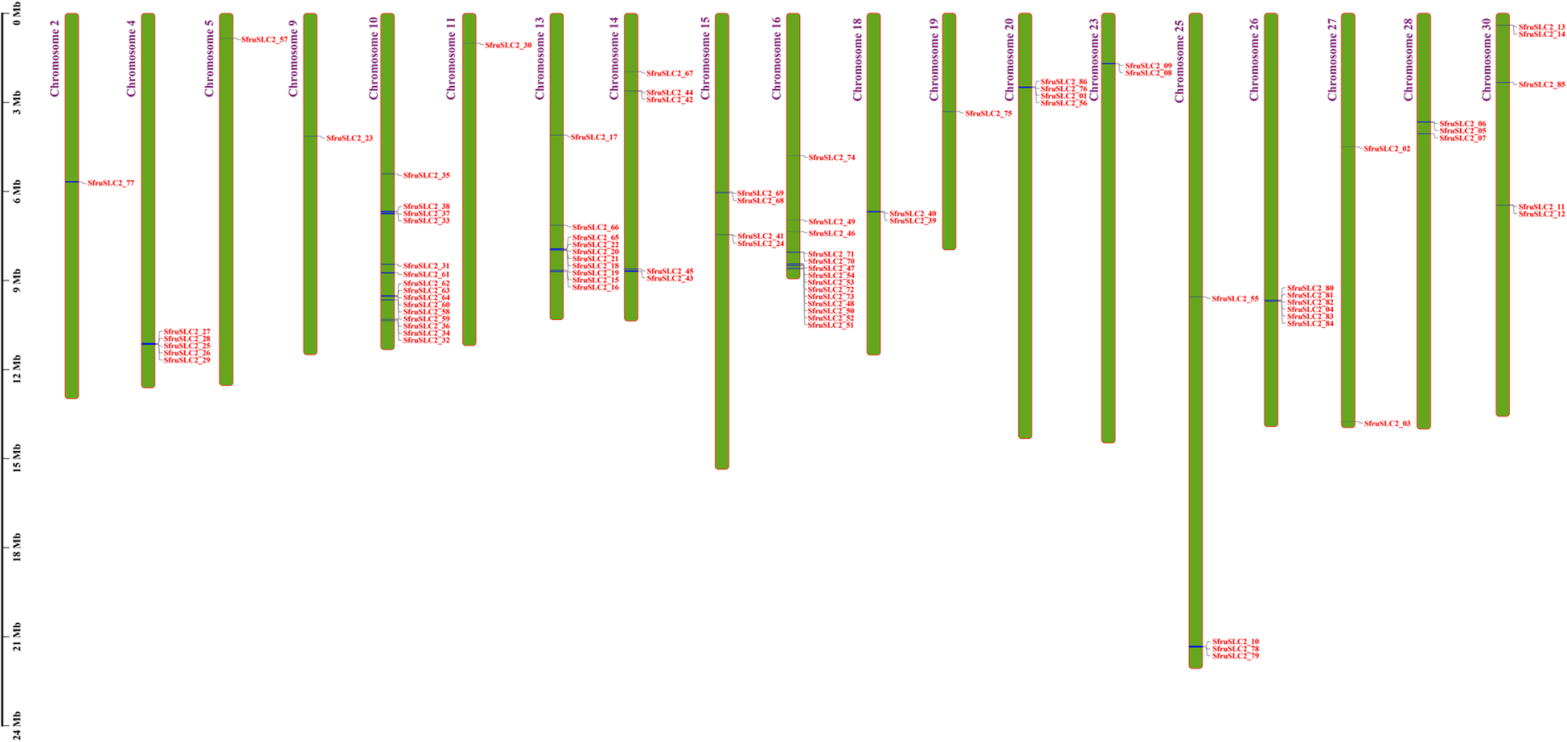
Chromosomal distribution of the *SfruSLC2* genes on 19 of the 31 chromosomes of *S. frugiperda*

The 86 *SfruSLC2* genes were distributed across 19 of the 31 *S. frugiperda* chromosomes (Fig. 11). Chromosome 10 contained the highest number (15 genes: *SfruSLC2_31–38, and 58–64*), whereas chromosomes 2, 5, 9, 11, and 19 each carried a single gene (*SfruSLC2_77, 57, 23, 30*, and *75*, respectively). The remaining 66 genes were unevenly distributed (2–14 genes) among the other 13 chromosomes.

**Fig.12:**
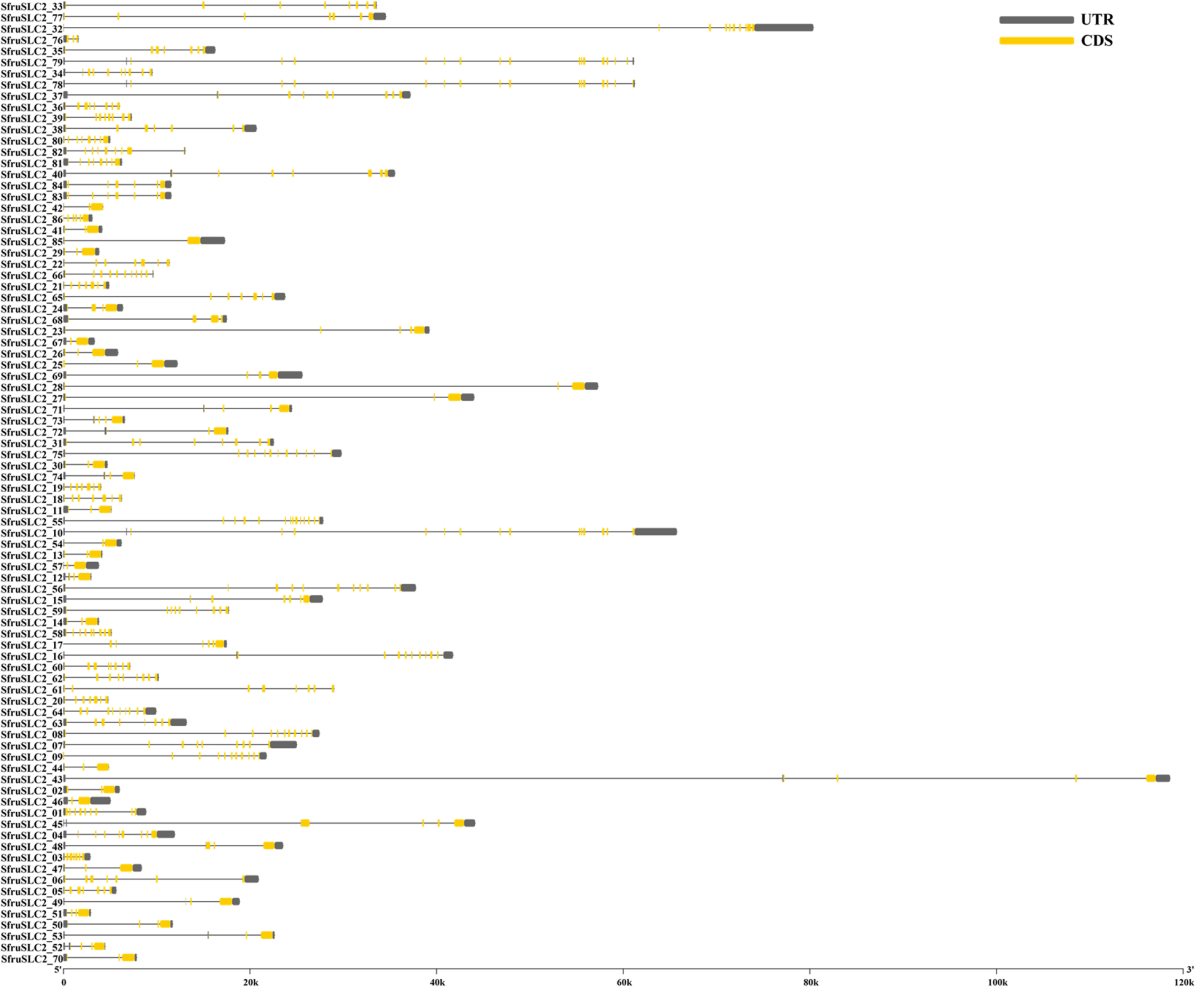
Gene structure visualization of the *SfruSLC2* genes. The gray color represents the untranslated regions (UTR) or Introns, and the yellow color represents the coding sequences (CDS) or Exons

The *SfruSLC2* gene structures were analyzed using the *S. frugiperda* genome (Fig. 12). The genes showed variable numbers of exons (2–18) and introns (2–15). *SfruSLC2_85* had the fewest exons (2), while *SfruSLC2_78* and *SfruSLC2_79* had the most (15). The longest gene was *SfruSLC2_43*, and the shortest was *SfruSLC2_76*. Except for *SfruSLC2_17* and *SfruSLC2_86*, all genes began with a 5′ UTR and ended with a 3′ UTR.

### Mature miRNAs of *SfruSLC2* CDSs

The 161 *S. frugiperda* miRNAs (Sfru-miRs) from InsectBase 2.0 were targeted against the 86 *SfruSLC2* CDS sequences. A total of 77 miRNAs belonging to 61 families were predicted to target 58 *SfruSLC2* mRNAs (Supplementary Table S7). Among them, *Sfru-miR-980* targeted the highest number of genes (*SfruSLC2_18, 23, 57, 63, 74*, and *81*). The longest miRNAs were *Sfru-miR-190* and *Sfru-miR-998* (24 bp each). *SfruSLC2_32* was the most highly targeted mRNA (7 miRNAs), while 18 mRNAs were targeted by only one miRNA. The remaining 38 mRNAs were regulated by 2–6 different miRNAs each.

### Insect laboratory and field treatments

**Fig. 13:**
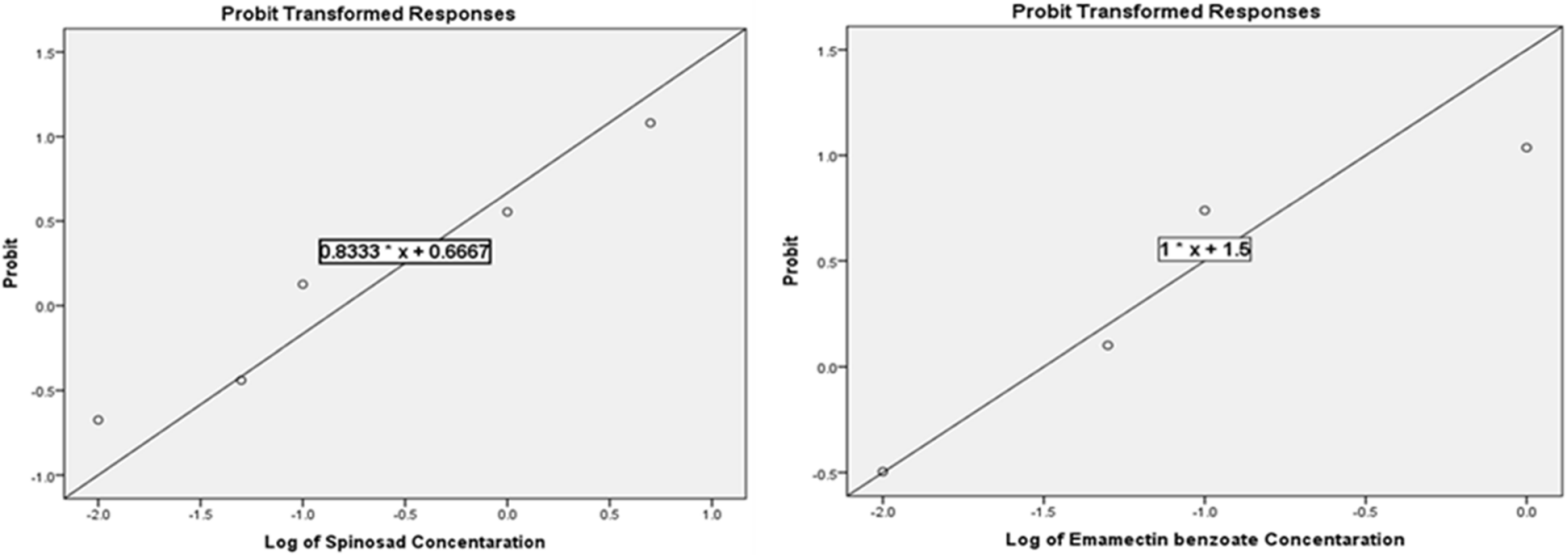
Toxicity regression lines of SPD and EMB against third instar larvae of *S. frugiperda* based on probit analysis, (SPD: y = 0.8333x + 0.6667, χ^2^ = 1.306, *p* = 0.728 & EMB: y = 1.000x + 1.500, χ.^2^ = 0.286, *p* = 0.867)

Laboratory bioassays (Fig. 13) revealed that both Ac-diluted SPD (Ac-SPD) and EMB (Ac-EMB) caused dose-dependent mortality in *S. frugiperda* larvae, with Ac-EMB being markedly more toxic. Probit analysis confirmed a good fit of the data (Ac-SPD: χ^2^ = 1.306, *p*-value = 0.728; Ac-EMB: χ^2^ = 0.286, *p*-value = 0.867), showing linear concentration–mortality relationships (Ac-SPD: y = 0.8333x + 0.6667; Ac-EMB: y = 1.000x + 1.500). LC₅₀ values indicated higher potency of EMB (0.031 ppm) than SPD (0.12 ppm), and LC₂₅ values (0.004 vs. 0.01 ppm, respectively) supported greater efficacy at sublethal doses, although both shared the same LC₁₀ (0.001 ppm). Acetone alone induced behavioral stress (reduced mobility, feeding inhibition, and delayed recovery) without early mortality, yet prolonged exposure caused delayed lethal effects that intensified with higher concentrations, more severe than with acetone–insecticide mixtures. Under semi-field conditions, both insecticides achieved 100% mortality of 2nd- and 4th-instar larvae within 24 h, with population suppression confirmed by the Henderson–Tilton formula.

### Expression analysis of *TRET* genes under stress conditions

**Fig. 14:**
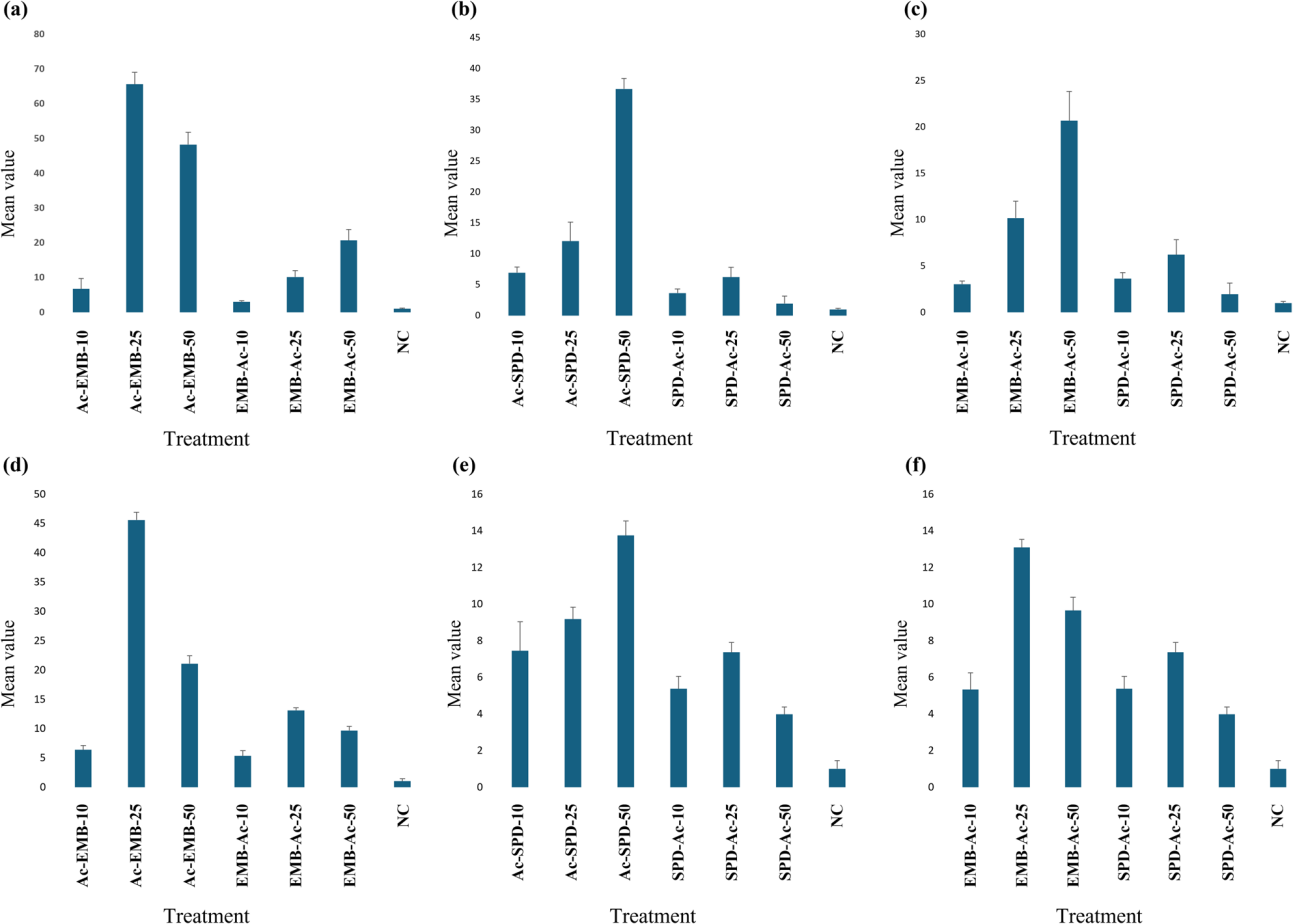
qRT-PCR analysis of *Tret1* and *Tret1-like* gene expression represented as fold change geometric mean on y-axis (mean ± SE, n = 3) and different treatments on x-axis; *Tret1* responses to EMB, SPD, Ac, and NC **a–c**, and *Tret1-like* responses to the same treatments **d–f**

**Fig. 15:**
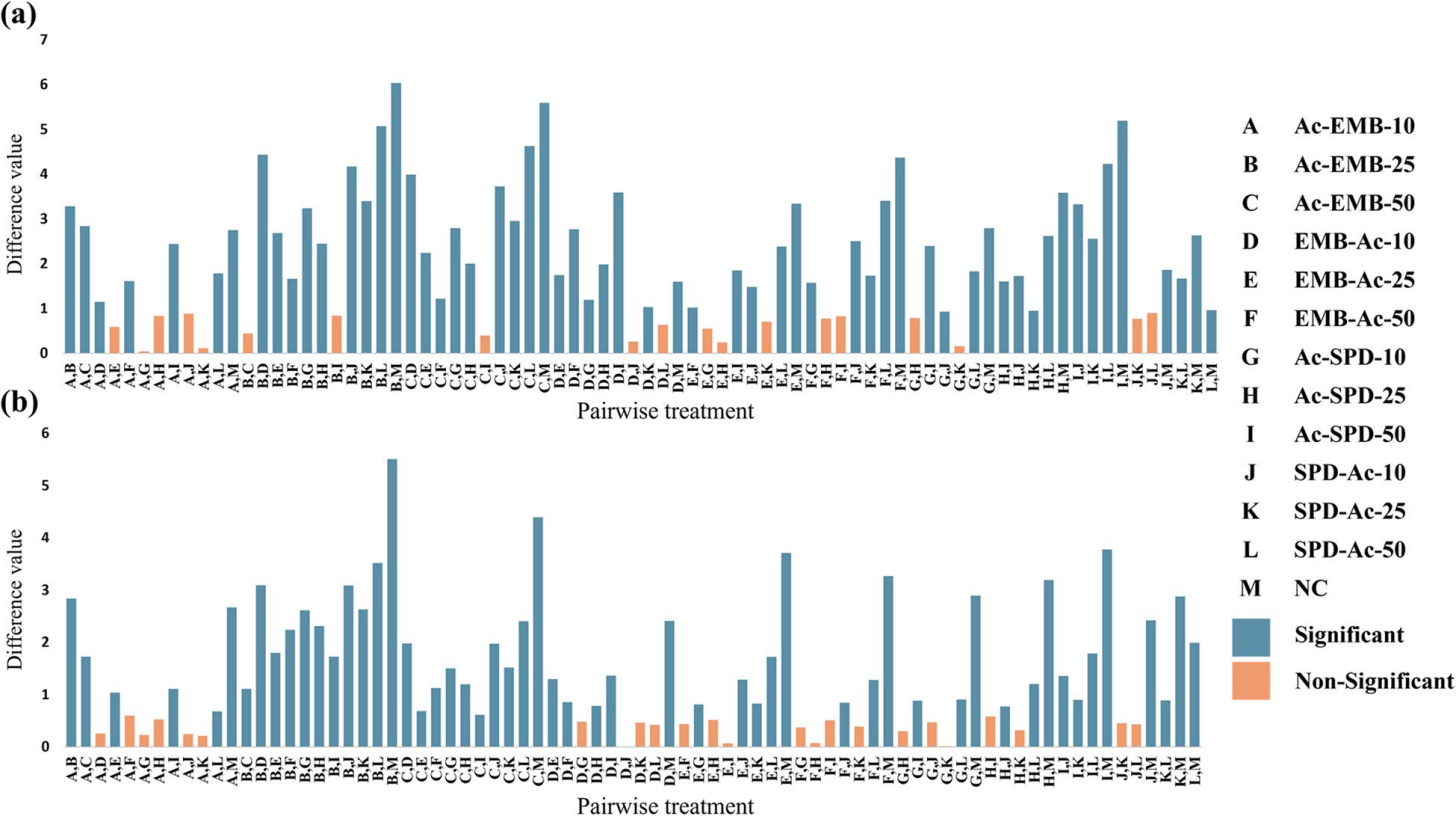
LSD post hoc test results of *Tret* gene expression responses determined by qRT-PCR Expression patterns of *Tret1* **a** and *Tret1-like* **b** under different treatments (EMB, SPD, and Ac), each applied at three sublethal concentrations (labeled A–L) along with the negative control (M). Bars represent pairwise differences between treatment means, where orange and blue indicate non-significant and significant differences, respectively (*p* < 0.0001)

Quantitative RT-PCR analysis was performed to evaluate the expression levels of *Tret1* and *Tret1-like* genes under different treatments, including the acetone-diluted technical forms of EMB (Ac-EMB) and SPD (Ac-SPD), pure acetone (Ac), and the negative control (NC) (Supplementary Table S8 and Fig. 14). Data were analyzed using one-way ANOVA followed by an LSD post-hoc test (Fig. 15). The ANOVA results revealed highly significant differences among treatments for both *Tret1* (*p*-value = 7.78 × 10⁻^13^) and *Tret1-like* (*p*-value = 1.32 × 10⁻^13^), both far below 0.0001 significance *p-*value threshold, indicating that exposure to insecticides and acetone markedly affected gene expression. The LSD test further showed that all treatments were significantly different from the negative control, whereas only some treatments differed significantly from each other, as indicated by the grouping letters in Fig. 15.

For *Tret1*, Ac-EMB exposure induced a non-linear response, with expression peaking at LC₂₅, then slightly declining at LC₅₀ but remaining above LC₁₀ levels (Fig. 14a). Ac related to EMB (EMB-Ac-10, 25, 50) treatments showed a similar trend but with lower induction. Ac-SPD caused linear upregulation across concentrations, while its related Ac treatments (SPD-Ac LCs) exhibited a non-linear pattern, also peaking at LC₂₅ (Fig. 14b). Among all treatments, Ac-SPD and Ac-EMB induced stronger upregulation than their corresponding acetone-only exposures, with significant differences between most LCs ( Fig. 15a). Acetone alone caused mild, concentration-dependent effects (Fig. 14c). Slight upregulation occurred at Ac-10 for both insecticides, increasing with EMB-Ac-25 and 50 but declining with SPD-Ac-25 and 50. Thus, EMB-Ac-50 appeared to represent a threshold concentration beyond which acetone may begin to suppress *Tret1*expression.

For the *Tret1-like* gene, the overall expression pattern closely resembled that of *Tret1*, except under the EMB-Ac treatments, which exhibited a non-linear trend (Fig. 14d–f). Among all treatments, Ac-EMB-25 caused the highest upregulation of the target gene, and the Ac-EMB treatment generally had the strongest upregulating effect on the *Tret1-like* gene. While acetone alone upregulated *Tret1-like* expression, its effect was weaker than that of Ac-SPD and Ac-EMB, with the latter consistently inducing the strongest response. Significant differences were observed between most Ac-EMB and EMB-Ac concentrations, except at LC₁₀, where both treatments showed similar effects (Fig. 15b). In contrast, Ac-SPD and SPD-Ac treatments showed inconsistent differences across the tested concentrations, with some levels exhibiting significant variation, while others did not. Moreover, all counterpart LCs were significantly different, except for LC₁₀, whose counterparts were generally non-significant across all treatments. Overall, acetone treatments produced a non-linear, concentration-dependent response, with significant differences among most LCs (Fig. 15b).

## Discussion

Our analysis identified 267 putative SfruMFS and 86 SfruSLC2 members, expanding the known repertoire of MFS transporters in insects, previously reported only in aphids [36] and *D. melanogaster* [3]*.* The number of SfruSLC2s was comparable to that in *B. mori* (88 members) but higher than in *D. melanogaster* (31 members) [2]. SfruSLC2 could be phylogenetically divided into three groups: GLUTs, TRETs, and Mixed Transporters. However, the *SLC2* gene family has not been categorized with a specific number of members in insect species [37]. The GLUT group included seven putative members, mainly GLUT1, GLUT1-like, and GLUT3. Their reduced number may be due to insects having evolved from ancestors with a limited number of GLUT copies [38], which are proposed to be sufficient for performing basic insect functions [39]. Therefore, insects may rely more on disaccharides such as trehalose rather than on glucose, making TRETs more essential than other GLUTs [40, 41]. Accordingly, TRETs formed the largest group (61 members), highlighting their role in trehalose homeostasis, consistent with previous reports [7, 12], emphasizing trehalose’s metabolic importance in insects. The mixed group contained 14 TRETs and four unclassified SLC2s that likely belong to the TRET subfamily according to their high bootstrap values and domain search, bringing the total to 79 members. Comparative phylogeny showed that Lepidopterans possess more *SLC2* genes than Dipterans, with TRETs forming distinct Dipteran-specific, Lepidopteran-specific, and shared subclades, whereas GLUTs were conserved across both orders. This finding reflects insects’ greater reliance on trehalose transport compared to glucose via GLUTs. Consequently, this pattern suggests that gene duplication and diversification of insect TRETs have resulted in multiple paralogs with distinct kinetic and tissue-specific properties [42].

The physicochemical properties of SfruSLC2 proteins revealed pI values ranging from 5.04 to 9.66, with 86.04% exhibiting alkaline characteristics due to the abundance of basic residues (Arg, Lys, and His). This overall positive charge likely contributes to the electrostatic stabilization of these proteins within the plasma membrane [43]. Most proteins (97.67%) had positive GRAVY values, confirming their hydrophobic, membrane-associated nature [44]. High aliphatic index (AI) values indicated thermostability driven by aliphatic residues (Ala, Val, Ile, Leu), which enhance the hydrophobic core crucial for transporter stability and function [11]. Moreover, 58.14% of proteins showed an instability index (II) below 40, suggesting overall structural stability consistent with their role as membrane transporters under variable physiological conditions [45–47].

The CDD search confirmed the integration of all putative SfruSLC2 proteins within the SLC2 family. Motif analysis revealed that both TRET and GLUT groups shared conserved motifs forming the main domain region, consistent with their phylogenetic classification. Notably, the motif pattern suggested the occurrence of mutation events among SfruSLC2 members. Most proteins exhibited random motif loss, sometimes leading to domain shortening that may indicate functional modification. In contrast, motifs 13 and 14 appeared in only four sequences, where additional N-terminal motifs in three of them implied a gain of function. Motif duplication or substitution events were rare, suggesting that SfruSLC2 diversification mainly involved loss rather than expansion. Collectively, such motif variability may reflect adaptive divergence, where selective pressure drives specialization or alteration of ancestral functions [48].

All SfruSLC2 proteins were localized to the plasma membrane, confirming their proposed function as transmembrane transporters [49]. Meanwhile, another highlighted subcellular location was the endoplasmic reticulum, which served as a secondary localization site for SfruSLC2, indicating their potential role in regulating protein translocation to the membrane and in membrane trafficking [50]. Other predicted subcellular localizations may integrate SfruSLC2 into additional, yet undefined, specific functions.

All SfruSLC2 members exhibited between 5 and 12 TMHs, suggesting that they are multipass integral membrane proteins. Interestingly, most MFS sugar transporters typically contain 12 TMHs [51], supporting the findings of this analysis. The SP is a short N-terminal amino acid sequence that directs proteins to the secretory pathway [52]. Only 12 SfruSLC2 members possessed SPs, indicating that the majority are non-secreted proteins whose intrinsic hydrophobicity likely serves as a membrane-targeting signal without the need for a signal peptide [53]. The few members containing SPs may represent secreted proteins with an N-terminal signal sequence.

Secondary and tertiary structural analyses of SfruSLC2 proteins revealed that α-helices were the dominant structural component. The prevalence of α-helices facilitates membrane spanning, allowing stable integration of the transmembrane transporter within the hydrophobic lipid bilayer and enabling the formation of pores essential for sugar transport [54].

SCMs play essential roles in maintaining the structure and function of SLC2 transporters across organisms [55]. In SfruSLC2, the predominant SCM was QL/FS in TM7. In GLUTs, this motif likely contributes to glucose selectivity over fructose, while its substitution (L → F) in some TRETs may confer trehalose specificity, though this remains unconfirmed [56]. The GRR/K motif, common in SfruSLC2s, is associated with conformational stability and transmembrane topology [7]. Additionally, PES/TP (in 71 members, mainly at the end of TM6) and PETK/RGK/R (in 40 TRETs) are implicated in substrate transport and ligand binding [37, 57].

The GO enrichment analysis supported the results of other characterization methods, as all SfruSLC2 proteins were associated with membrane cellular components and the transmembrane transport biological process, which involves the movement of solutes across the lipid bilayer. Additionally, the carbohydrate transport process was enriched, describing the directed movement of carbohydrates into, out of, or within cells via transporters or pores. The predicted molecular functions of SfruSLC2s included transmembrane transporter activity and sugar transmembrane transporter activity, reflecting their role in transferring specific substances, particularly monosaccharides and small oligosaccharides across cellular membranes [58].

The PPI network analysis revealed that only 23 SfruSLC2 proteins formed the main interaction network, suggesting that these represent the functionally active or co-expressed subset within the family. Among them, SfruSLC2_55 acted as a central hub with 22 direct interactions, highlighting its potential as a key functional regulator. A tightly connected subnetwork comprising SfruSLC2_15, SfruSLC2_24, SfruSLC2_41, and SfruSLC2_77 formed part of the main network, indicating a cooperative functional module likely involved in related physiological pathways. In contrast, an independent cluster (SfruSLC2_08, SfruSLC2_09, and SfruSLC2_53) exhibited few interactions and no connection to the core hub, implying that these proteins may function in distinct or tissue-specific regulatory contexts.

The 86 *SfruSLC2* genes were distributed across 19 of the 31 *S. frugiperda* chromosomes. Most genes were clustered on specific chromosomes, suggesting possible tandem duplication events [59]. Gene structure analysis showed typical exon–intron organization. *SfruSLC2_85* had the fewest exons and introns (two each), implying a simple, broadly expressed gene, whereas *SfruSLC2_78* and *SfruSLC2_79* contained numerous exons and introns, indicating higher complexity and potential alternative splicing, which may expand functional diversity [60]. *SfruSLC2_43* was the longest gene, possibly reflecting extensive regulatory control, while *SfruSLC2_76* was the shortest, with unclear functional implications. All genes contained a 5′ UTR, except *SfruSLC2_17* and *SfruSLC2_86*, suggesting these may be pseudogenes. Conversely, the presence of a 3′ UTR in all members supports roles in mRNA stability and translational efficiency [61].

Targeting of *SfruSLC2* CDSs by Sfru-miRs revealed 77 miRNAs belonging to 61 families, reflecting the broad regulatory network controlling *SfruSLC2* functions. *Sfru-miR-980* targeted the largest number of genes and is known to regulate memory suppression and neuronal excitability [62]. Some transcripts, such as *SfruSLC2_32*, were targeted by multiple miRNAs (seven in total), suggesting these genes play essential or multifunctional roles [63]. In contrast, several *SfruSLC2* mRNAs were targeted by only one miRNA, indicating specific regulation where a single miRNA may suffice for expression control. Overall, these findings emphasize that Sfru-miRs likely fine-tune sugar transporter activity through miRNA–mRNA interactions, influencing glucose and carbohydrate uptake at the cellular level [64].

Laboratory bioassays on *S. frugiperda* larvae revealed that both Ac-SPD and Ac-EMB were effective, though Ac-EMB showed markedly higher potency. Ac-EMB’ LC₅₀ (0.031 ppm) was approximately fourfold lower than that of Ac-SPD (0.12 ppm), and its LC₂₅ (0.004 ppm) was 2.5 times lower than that of Ac-SPD (0.01 ppm) indicating that Ac-EMB achieves stronger control at lower doses, supporting its suitability for IPM programs [31, 65, 66]. Both mixtures shared the same LC₁₀ (0.001 ppm), suggesting that at very low concentrations, both compounds initiate early mortality with similar efficacy [67]. The higher toxicity of EMB likely results from its action on glutamate-gated chloride channels, causing paralysis and death [68], whereas SPD acts on nicotinic acetylcholine receptors, inducing hyperexcitation of the nervous system and muscle fatigue [69]. While effective, SPD generally requires higher concentrations to achieve comparable mortality [67]. Additionally, Ac treatments induced mild stress in larvae, consistent with previous findings suggesting that certain solvents, including acetone, can enhance insect mortality or alter neurophysiological responses [70, 71]. These findings suggest that acetone may play a role in enhancing the insecticidal effect of formulated mixtures or altering insect behavior independently.

Semi-field trial under plastic tunnels using staked tomato plants confirmed the high efficacy of both SPD and EMB against *S. frugiperda* larvae. At recommended field rates, both insecticides achieved 100% mortality within 24 h in second- and fourth-instar larvae, consistent with laboratory results [72–74]. Population reduction calculated by the Henderson–Tilton formula [75] further validated their performance under near-natural conditions. The rapid and consistent action particularly of EMB, which exhibited lower LC values supports their potential use in IPM programs for tomato pest management. Nevertheless, rotation with other insecticide classes is essential to mitigate resistance risks.

Gene expression analysis revealed a marked upregulation of *TRET* genes in larvae treated with Ac-EMB and Ac-SPD complexes, indicating a strong physiological response to the neurotoxic stress induced by both insecticides. As these compounds disrupt neural transmission and feeding activity [31, 76], the resulting nutritional deficit triggered the mobilization of internal energy reserves, Therefore, elevated *TRET* expressions may reflect an adaptive mechanism to maintain trehalose supply for essential detoxification and survival processes [11, 77]. The differential magnitude of *TRET* gene responses between Ac-EMB and Ac-SPD reflected their distinct modes of action. The stronger and more persistent neurotoxic effect of EMB likely triggered a more intense stress response, explaining the higher expression levels compared to SPD. Notably, the non-linear dose–response pattern of Ac-EMB, where gene expression declined at very high concentrations, supports the “overwhelm effect” hypothesis, suggesting that severe toxicity prevents the activation of a complete molecular response. However, it should be noted that this interpretation remains hypothetical and would require further biochemical or metabolite evidence (e.g., trehalose levels or enzyme activities) to confirm such an “overwhelm effect” at the physiological level. In contrast, SPD exhibited a linear response, consistent with its moderate potency and slower mode of action. Despite EMB’s superior toxicity, SPD may still be favored in some agricultural contexts due to its natural origin and lower environmental persistence [65, 72, 78]. Interestingly, Ac alone induced *TRET* genes upregulation, indicating ingestion-related physiological stress, which consists of previous reports about the biologically non-neutral nature of acetone varies depending on the method of application [70, 79–82]. The non-linear dose–response pattern observed with Ac also aligned with the "overwhelm effect" hypothesis, which should likewise be interpreted cautiously until verified by biochemical assays, suggesting this is a general mechanism for potent toxic agents. The combined Ac–insecticide treatments elicited a markedly stronger response, suggesting a possible synergistic interaction where Ac may enhance insecticide absorption and bioavailability within the larval gut [83–85]. Overall, the gene expression patterns of *TRET* genes were consistent with the physiological stress and mortality observed in both laboratory and semi-field bioassays, emphasizing the potential metabolic and developmental impacts of EMB, SPD, and Ac under the tested conditions [16].

## Conclusion

The current study identified 267 putative SfruMFS members, including 86 SfruSLC2 proteins that were fully characterized. Phylogenetic analysis classified SfruSLC2 into three groups: GLUTs, TRETs, and mixed transporters (likely TRETs). Notably, the TRET subfamily exhibited greater divergence among different insect species compared to the more conserved GLUT subfamily, suggesting that TRETs may play a crucial role in insect adaptation to diverse environmental and physiological stresses. The physicochemical properties and conserved domains confirmed their identity as stable, membrane-associated sugar transporters. Motif analysis indicated random motif loss in several members, which may reflect structural adaptation or novel functional diversification. Most SfruSLC2 proteins localized to the plasma membrane and contained 5–12 transmembrane helices, consistent with their role as multipass integral membrane proteins. Only 12 proteins contained signal peptides, suggesting that most are non-secretory. Structural modeling showed α-helices as the dominant component, supporting their membrane-embedded function. SCMs analysis distinguished SfruSLC2-TRETs and SfruSLC2-GLUTs, reinforcing their substrate specificity. GO annotation confirmed roles in transmembrane carbohydrate transport, while the PPI network identified SfruSLC2_55 as a potential key regulatory hub. Chromosomal mapping revealed that the 86 *SfruSLC2* genes are distributed across 19 of the 31 chromosomes, often in tandem clusters. Gene structure analysis showed typical exon–intron organization, and miRNA targeting suggested multilayered post-transcriptional regulation involving 77 miRNAs from 61 families. Laboratory and semi-field bioassays demonstrated that EMB exhibited markedly higher potency than SPD at lower LC values, indicating a stronger toxic effect but also a greater risk for resistance development. Acetone alone induced mild physiological stress and, when combined with insecticides, significantly enhanced toxicity, as confirmed by *TRET* gene expression patterns. The non-linear upregulation trends observed for EMB and Ac contrasted with the linear response to SPD, supporting the hypothesis of differential stress regulation. Overall, these findings provide the first comprehensive characterization of the *SfruSLC2* gene family and highlight their potential role in stress adaptation and energy metabolism under insecticidal exposure. Alternating between SPD and EMB, or integrating them with other control strategies, could improve long-term pest management while minimizing resistance. Importantly, the biological role of acetone in modulating insecticidal effects warrants further investigation. Finally, this study provides the first comprehensive genome-wide characterization of the *SLC2* gene family in *S. frugiperda*, distinguishing its members into two major subfamilies (TRETs and GLUTs) and a mixed clade. This refined classification extends the current understanding of *SLC2* gene diversity beyond previous reports in other lepidopterans. Moreover, the demonstrated transcriptional responses of *TRET* genes to the bioinsecticides highlight their potential roles in mediating physiological and toxicological adaptation in this pest species.

## Material and methods

### Identification of SfruMFS putative members

A Hidden Markov Models (HMMs) of the MFS conserved domains (PF07690, PF16983, PF05631, PF07672, PF05977, PF06779, PF12832, and PF13347) were downloaded from the Pfam database that is hosted by InterPro—EMBL-EBI [86] and used to search for MFS sequences in the reference proteome of *S. frugiperda* (GCF_023101765.2) using HMMER v3.3.2 (E-value < 10⁻^5^) to detect putative sequences. The presence of MFS domains in the resulting sequences was investigated using InterPro, the protein sequence classification resource [87], and sequences lacking these conserved domains were eliminated. Redundant sequences (≥ 97% similarity) were eliminated using the CD-HIT tool [88] with a sequence identity threshold of 0.97. For more assurance, identified MFS protein sequences were aligned to MFS protein sequences downloaded from the *D. melanogaster* database [89] using the protein basic local alignment search (BLASTP) online tool [90], with an E-value cutoff of 1e − 5 and default parameters for scoring matrix and gap penalties.

### Phylogenetic analysis and characterization of potential SfruSLC2 members

To verify *S. frugiperda* SLC2 (SfruSLC2) members, the *D. melanogaster* SLC2 proteins (DmelSLC2; FBgg0000691) were retrieved from *D. melanogaster* Database [89] and aligned with identified SfruMFS proteins using MAFFT version 7 [91]. A phylogenetic tree was constructed using FastTree v2.1.11 [92], with 1000 bootstrap replications and the neighbor-joining algorithm under the Jones–Taylor–Thornton (JTT) model. SfruMFS proteins included in the same subclade with DmelSLC2 represented the verified SfruSLC2, which were subsequently introduced to phylogenetic analysis to identify their classification and relationships. MUSCLE v5.2 [93] was used to perform multiple sequence alignment, and the phylogenetic tree was built using the same method applied previously. To discover the evolutionary relationship between verified SfruSLC2 and other lepidoptera and non-lepidoptera SLC2 members, the SLC2 protein sequences of *Spodoptera litura*, *Bombyx mori*, *Bombyx mandarina*, *Bicyclus anynana*, *Galleria mellonella*, *Papilio machaon*, *Trichoplusia ni*, and *Helicoverpa armigera* were retrieved from the National Center for Biotechnology Information (NCBI) [94] to be used as lepidoptera SLC2 members, while DmelSLC2 proteins were used as non-lepidoptera (Diptera) SLC2 members. Multiple sequence alignment was performed using MAFFT v7 [91]. Phylogenetic analysis was conducted in IQ-TREE v2 [95] employing the maximum likelihood (ML) algorithm with 1,000 ultrafast bootstrap replicates to ensure statistical robustness. The best-fit substitution model was selected automatically using the TEST mode in IQ-TREE based on the Bayesian Information Criterion (BIC), which identified the JTT model as optimal for the dataset.

All phylogenetic trees were edited using the interactive Tree Of Life (iTOL) v6 [96]. The Expasy online resource [97] was used to clarify the protein length, MW, theoretical PI, GRAVY, AI, II, and positively and negatively charged residues for the SfruSLC2 proteins.

### Domain architecture and motif analyses of SfruSLC2 proteins

The NCBI Batch Web CD-Search tool [98] was used to search for SfruSLC2 conserved domains using the default parameters, except for the database, which was determined as CDD-62456 PSSMs. The conserved motifs of the SfruSLC2 sequences were analyzed using the MEME SUIT online tool [99] with default parameters except 20 motifs as the maximum number of motifs. The domains and conserved motifs were visualized using TBtools v2.210 [100] desktop software.

### Subcellular localization, signal peptide, and transmembrane structure analyses of SfruSLC2 family

SfruSLC2 subcellular localizations were verified using the WOLF PSORT online tool [101], and the resulting data was then illustrated as a heatmap using TBtools. Phobius online tool [102] was used to predict the number of transmembrane domains and the signal peptides within the SfruSLC2 proteins.

### Secondary and tertiary structure analyses of SfruSLC2 proteins

The SOPMA online tool, hosted by Network Protein Sequence Analysis [103], was used to predict the secondary structure of SfruSLC2 proteins, applying the default parameters, except that the number of conformational states was set to 4. The SWISS-MODEL online tool hosted by Expasy [104] was used to predict the SfruSLC2 proteins’ tertiary structures, then evaluated using the QMEANBrane scoring function implemented in its assessment tool.

### Short conserved motif analysis of SfruSLC2

The FIMO scans (v5.5.8) online tool [105] was used to predict the SLC2 function–related conserved motifs (QL/FS, PES/TP, GRR/K, PET, and PETK/RGK/R) [7]. Subsequently, the WebLogo (v2.8.2) online tool [106] was used to generate motif logos. The locations of these conserved motifs were determined in the previously detected SfruSLC2 transmembrane structures. The SOSUI online tool [107] was used to visualize the conserved motifs on the SfruSLC2 secondary structure.

### Gene ontology and protein–protein interaction analyses of SfruSLC2s

InterPro, the protein sequence classification resource [87] was used to classify gene ontology for each SfruSLC2 protein. SfruSLC2 proteins and the proteome of *S. frugiperda* (GCF_023101765.2) downloaded from NCBI were uploaded to the STRING database [108] to obtain the SfruSLC2 protein–protein interaction network.

### Chromosomal localization and gene structure analyses of *SfruSLC2* genes

*SfruSLC2* genes’ carrier chromosomes and start–end positions were obtained from NCBI, and their general feature formats were obtained from the General Feature Format Version 3 (GFF3) file belonging to *S. frugiperda* (GCF_023101765.2), which was also downloaded from NCBI. The obtained data were introduced to TBtools v2.210 to visualize the chromosome location map of the *SfruSLC2* gene family and its gene structure, respectively.

### Prediction of mature miRNAs targeting *SfruSLC2* genes

To characterize transcriptional regulation of the *SfruSLC2* genes, the mature miRNA sequences of *S. frugiperda* were retrieved from the InsectBase 2.0 database [109] and then targeted to the coding sequences (CDS) of the identified *SfruSLC2* genes by psRNATarget online tool [110].

### Insect materials and experimental treatments

Larvae of the fall armyworm, *S. frugiperda* (J.E. Smith) (Lepidoptera: Noctuidae), were collected during the summer of 2024 from infested maize fields located in Giza Governorate, Egypt. The collected larvae were reared in the laboratory for four generations under controlled conditions (25 ± 2 °C, 70 ± 10% RH, and a photoperiod of 14:10 h light: dark). Larvae were fed daily on freshly collected castor bean leaves (*Ricinus communis* L.) obtained from pesticide-free areas in the same region. Castor bean leaves were selected as the larval diet because they are more palatable and suitable for *S. frugiperda* larvae compared to maize or tomato leaves, and they are also readily available throughout the year. Leaves were washed with distilled water and air-dried before use. Adult moths were housed in 30 × 30 × 20 cm glass cages covered with fine mesh. A cotton pad soaked in a 10% sucrose solution was provided as a carbohydrate source. Egg-laying substrates (folded paper) were placed in the cages and collected daily.

Two insecticides were used in this study, with both the Technical Grade (TC) and the Commercial Formulation being evaluated for each. The first insecticide, designated EMB, has an active ingredient purity of 95% TC, and its commercial product is Speedo® 57% WG (Wettable Granules). The second, designated SPD, has an active ingredient purity of 92% TC, and its commercial formulation is Master Top® 48% SC (Suspension Concentrate). All insecticides were obtained from Star Chem Co., Egypt.

For the laboratory bioassays, a 1000 ppm stock solution (1 mg/ml) was prepared for each insecticide. This involved dissolving 0.1 g of the TC in 0.25 ml Ac for EMB and 0.5 ml for SPD. The dissolved material was then diluted with distilled water to a final volume of 100 ml to achieve the desired concentration. Subsequently, serial dilutions were performed to obtain the working concentrations, which were strategically selected based on the recommended field rate (80 g/200 L and 40 cm^3^/200 L) of commercial formulation of EMB and SPD, respectively as specified by the World Health Organization [111].

Lethal concentrations (LC₁₀, LC₂₅, and LC₅₀) of EMB and SPD were determined through laboratory bioassays using third-instar larvae. Probit analysis was performed using IBM SPSS Statistics (version 20). The calculated LC values for EMB were 0.001 ppm (LC₁₀), 0.004 ppm (LC₂₅), and 0.031 ppm (LC₅₀), while those for SPD were 0.001 ppm, 0.01 ppm, and 0.12 ppm, respectively. To assess any potential toxic effect of the solvent (Ac) alone, corresponding Ac concentrations equivalent to the LC values were tested separately. For EMB, the solvent concentrations were 2.5 × 10⁻⁷ % (LC₁₀), 1.0 × 10⁻⁶ % (LC₂₅), and 7.75 × 10⁻⁶ % (LC₅₀). For SPD, they were 5 × 10⁻⁷ % (LC₁₀), 5 × 10⁻⁶ % (LC₂₅), and 5 × 10⁻^5^% (LC₅₀). Treatment with distilled water was also included as NC. All treatment and control solutions were freshly prepared before each trial.

The bioassays were conducted under the same environmental conditions as the rearing phase. For each concentration and control, three replicates were used, each consisting of 10 healthy third-instar larvae placed in 300 ml glass jars. Before treatment, the larvae were starved for 6 h only to minimize the effect of cannibalistic behavior and to ensure that all individuals began feeding uniformly during the bioassay. Fresh castor bean leaves were dipped in each test solution for 30 s, then air-dried at room temperature for approximately 15 min. Treated leaves were introduced into the jars and replaced every 24 h. Larval mortality was recorded after 24 h of exposure. Larvae were considered dead if they failed to respond to gentle prodding with a fine brush. Mortality percentages were corrected using Abbott’s formula [112] to account for natural mortality in control groups. The experiment was repeated on three different days to confirm reproducibility.

A semi-field experiment was conducted under greenhouse conditions at the experimental station of the Faculty of Agriculture, Ain Shams University, using staked tomato plants (*Solanum lycopersicum L*.) at the flowering stage. Insecticide treatments were applied during early morning hours using a calibrated knapsack sprayer under calm environmental conditions. For each insecticide, 10 tomato plants were sprayed with the corresponding commercial formulation. To prevent spray drift and cross-contamination between treatments, untreated buffer rows were maintained between each treated group. Two hours after application, leaf samples were collected from the treated plants and transferred to the laboratory in ventilated containers for feeding assays, which targeted second and fourth-instar larvae. Six replicates per treatment were prepared, each consisting of 10 larvae placed in 300 ml glass jars containing treated leaf material. All jars were kept under controlled conditions (25 ± 2 °C, 60 ± 10% RH, and a 14:10 h light: dark photo period). Larval mortality was assessed after 24 h. The larvae were considered dead if they showed no response to gentle prodding with a fine brush. Field efficacy was assessed based on the reduction in larval populations using the Henderson and Tilton formula [75].

### *TRET* gene expression analysis

The response of *Tret1*and *Tret1-like* genes to insecticides dissolved in Ac (Ac-EMB and Ac-SPD), Ac only (3 per each insecticide; EMB-Ac-10, 25, 50 and SPD-Ac-10, 25, 50), and NC (Larvae received untreated castor bean leaves). laboratory treatments were quantified using real-time PCR. According to the manufacturer’s recommendations, total RNA was extracted from fresh treated larvae using ABT total and microRNA Mini Extraction Kit (Applied Biotechnology, Egypt). Extracted RNA was converted to cDNA using ABT 2X RT Mix oligo kit (Applied Biotechnology, Egypt). The qRT-PCR reactions were performed on a Stratagene MX3000 P (Agilent Technologies) machine using ABT 2X qPCR Mix, SYBR (Applied Biotechnology, Egypt). Primer pairs of *Tret1*(fwo: 5' GTGTTCACTGTGATGGCGGT 3' and rvs 5' GCCTGTGACACTCCCATGTC 3'), *Tret1-like* (fwo: 5' CCATCCCAAGCGAAGAGGTT 3' and rvs: 5' AACCATTGCGGACTCTCTGG 3'), and *EF1α*, an internal reference, (fwo: 5' GTCGCTGGTGACTCCAAGAA 3' and rvs: 5' TGATTTCGGCGAACTTGCAG 3') genes were designed depending on the NCBI Reference accessions of XM_035588915.2, XM_050693729.1, and XM_035588389.2 respectively. The Primer Blast online tool [113] was used to design the mentioned primers and verify their self-annealing and hairpin formation. The in-silico PCR tool [114] was used to validate the primer specificity. The primers were synthesized by Invitrogen, UK. All qRT-PCR data were obtained from triplicate samples. The relative expression levels of target genes were calculated by the 2^−ΔΔCt^ method [115] using Microsoft Excel 365 software. One-way analysis of variance (ANOVA) was used to assess differences among treatments, followed by the least significant difference (LSD) post-hoc by SPSS software. Differences were considered statistically significant at *p*-value < 0.0001.

## Supplementary Information

Supplementary Material 1

## Data Availability

The datasets generated and/or analyzed during the current study are included in this article and its supplementary information files.

## Abbreviations

a.a: Amino Acid
Ac: Acetone
Ac-EMB: Acetone-diluted Emamectin benzoate
Ac-SPD: Acetone-diluted Spinosad
AI: Aliphatic Index
ANOVA: Analysis of variance
BLASTP: Protein basic local alignment search online tool
BP: Biological Process
CC: Cellular component
CDD: NCBI's Conserved Domain Database
CDS: Coding sequences
CT: Cycle Threshold
DmelSLC2: *Drosophila melanogaster* SLC2
EMB: Emamectin benzoate
EMB-Ac-10: The amount of Ac used to dissolve EMB to produce a LC10
EMB-Ac-25: The amount of Ac used to dissolve EMB to produce an LC25
EMB-Ac-50: The amount of Ac used to dissolve EMB to produce an LC50
GFF3: General Feature Format Version 3
GLUTs: Glucose Transporters
GO: Gene Ontology
GRAVY: Grand Average of Hydropathy
HMMs: Hidden Markov Models
II: Instability Index
LC₁₀: Lethal concentration 10 that causes death in 10% of a test larva.
LC₂₅: Lethal concentration 25 that causes death in 25% of a test larva.
LC₅₀: Lethal concentration 50 that causes death in 50% of a test larva.
LSD: Least Significant Difference
MF: Molecular Function
MFS: Major facilitator superfamily
NC: Negative control
NCBI: National Center for Biotechnology Information
pI: Theoretical isoelectric point
PPI: Protein–protein interaction
qRT-PCR: Quantitative reverse transcription polymerase chain reaction
SE: Standard error
SfruMFS: *Spodoptera frugiperda* MFS
Sfru-miR: *S. frugiperda* MiRNA
*SLC*: Solute carrier gene superfamily
*SLC2*: Solute carrier 2 gene family
SPD: Spinosad
SPD-Ac-10: The amount of Ac used to dissolve SPD to produce an LC10
SPD-Ac-25: The amount of Ac used to dissolve SPD to produce an LC25
SPD-Ac-50: The amount of Ac used to dissolve SPD to produce an LC50
SPs: Signal peptides
TMHs: Transmembrane helices
*Tret1*: Facilitated trehalose transporter gene
*Tret2/Tret1-like*: Facilitated trehalose transporter-like gene
TRETs: Trehalose transporters subfamily
UTR: Untranslated regions
